# Synthesis and Evaluation of New Benzodioxole- Based Thiosemicarbazone Derivatives as Potential Antitumor Agents

**DOI:** 10.3390/molecules21111598

**Published:** 2016-11-22

**Authors:** Mehlika Dilek Altıntop, Halide Edip Temel, Belgin Sever, Gülşen Akalın Çiftçi, Zafer Asım Kaplancıklı

**Affiliations:** 1 Department of Pharmaceutical Chemistry, Faculty of Pharmacy, Anadolu University, 26470 Eskişehir, Turkey; belginsever@anadolu.edu.tr (B.S.); zakaplan@anadolu.edu.tr (Z.A.K.); 2 Department of Biochemistry, Faculty of Pharmacy, Anadolu University, 26470 Eskişehir, Turkey; heincedal@anadolu.edu.tr (H.E.T.); gakalin@anadolu.edu.tr (G.A.Ç.)

**Keywords:** thiosemicarbazone, benzodioxole, cancer, apoptosis, mitochondrial membrane potential, SIRT1, docking studies

## Abstract

New benzodioxole-based thiosemicarbazone derivatives were synthesized and evaluated for their cytotoxic effects on A549 human lung adenocarcinoma, C6 rat glioma and NIH/3T3 mouse embryonic fibroblast cells. In order to examine the correlation between anticancer activity and cholinesterases, the compounds were evaluated for their inhibitory effects on AChE and BuChE. The most effective anticancer agents were investigated for their effects on DNA synthesis, apoptosis and mitochondrial membrane potential. 4-(1,3-Benzodioxol-5-yl)-1-([1,1′-biphenyl]-4-ylmethylene)thiosemicarbazide (**5**) was identified as the most promising anticancer agent against C6 and A549 cell lines due to its inhibitory effects on C6 and A549 cells and low toxicity to NIH/3T3 cells. Compound **5** increased early and late apoptosis in A549 and C6 cells. Compound **5** also caused disturbance on mitochondrial membrane potential and showed DNA synthesis inhibitory activity in A549 and C6 cells. Compound **5** was investigated for SIRT1 inhibitory activity to provide mechanistic insight and for that purpose docking studies were also performed for this compound on SIRT1. On the other hand, compound **5** did not show any inhibitory activity against AChE and BuChE. This outcome pointed out that there is no relationship between anticancer activity of compound **5** and cholinesterases.

## 1. Introduction

Cancer is an abnormal cell division caused by some alteration in the expression of plentiful genes in the nucleus or in the mitochondria of cells. These alterations lead to a disorder in the balance between cell reproduction and cell death. Indeed, cancer is not a single disease, but rather it is a group of more than a hundred distinct diseases affecting different organs and systems of the body. Up to now, many chemotherapeutic drugs have been developed as anticancer agents, but most of them are insufficient for cancer treatment because of their lack of specificity towards cancer cells and correspondingly their high risk of toxicity to normal cells. Consequently, pharmaceutical research has focused on the discovery of more selective and less toxic anticancer agents [1,2,3,4,5,6].

Acetylcholinesterase (AChE) and butyrylcholinesterase (BuChE) are abnormally expressed in various pathological conditions, such as tumorigenesis. In recent years, AChE has attracted a great deal of interest as a potential therapeutic target for the treatment of cancer due to the involvement of this enzyme in apoptosis and cell adhesion, differentiation, and proliferation [7,8,9].

The mammalian sirtuins are a family of NAD^+^-dependent enzymes that have been connected to a great variety of biological activities encompassing cellular stress resistance, genomic stability, tumorigenesis and energy metabolism. Among them, sirtuin-1 (SIRT1) which belongs to the class III histone deacetylases (HDACs), is associated with a wide range of human diseases and is an outstanding potential therapeutic target. Overexpression of SIRT1 has been reported to increase growth and survival rates of some cancer cell lines and inhibit apoptosis [10,11,12,13]. However, the role of sirtuins as tumor suppressor or tumor promoter in some types of cancer is more diverse and complex than previously anticipated due to cellular context or targets in specific signaling pathways [14].

Thiosemicarbazones have been the subject of considerable research due to their wide range of biological activities such as antitumor, antibacterial, antifungal and antiviral effects. Since the 1950s, their anticancer activity has been continually investigated and some of them were explored as antileukemic agents. Thiosemicarbazones have iron coordination capacity and the complexation with most of the transition metal ions has increased their use as potential antitumor agents. The antitumor activity of thiosemicarbazones has mainly been associated with their inhibition capacity of ribonucleotide reductase, which is the rate limiting enzyme in the synthesis and repair of DNA. The ability of thiosemicarbazones to chelate metal ions has been defined as a major mechanism in the inhibition of ribonucleotide reductase. For this purpose, 3-aminopyridine-2-carboxaldehyde thiosemicarbazone (3-AP or Triapine^®^), a promising potent molecule, was studied in several clinical phase I and II trials where it was found to be ineffective against a variety of solid tumors but very promising against hematologic malignancies such as leukemia. Triapine^®^ was 1000 times more potent than the clinically used ribonucleotide reductase inhibitor hydroxyurea in both enzyme and cell assays. Recent studies have also showed that thiosemicarbazones inhibit topoisomerase-II α enzyme, which is highly expressed in many cancer cells acting by stabilization of the cleavable products formed by topoisomerase-II and DNA through a thiol alkylation. In the recent times, thiosemicarbazones were reported to inhibit ATP binding cassette (ABC) transporters, which are involved in the development of multidrug resistance [15,16,17,18,19,20,21,22,23]. On the other hand, 1,3-benzodioxole is found in a variety of anticancer agents such as podophyllotoxin, steganacin, and combretastatin A-2 showing good bioavailability and low cytotoxicity [24].

On the basis of aforementioned findings, in the present investigation we report the synthesis and evaluation of a new series of benzodioxole-based thiosemicarbazone derivatives as anticancer agents against A549 human lung adenocarcinoma, C6 rat glioma and NIH/3T3 mouse embryonic fibroblast (healthy) cell lines. The synthesized compounds were evaluated for their inhibitory effects on AChE and BuChE to examine the correlation between anticancer activity and cholinesterases. Furthermore, the most effective anticancer agents were also evaluated for their effects on DNA synthesis, apoptosis and mitochondrial membrane potential. The SIRT1 inhibitory action of compound **5**, the most promising anticancer agent, was investigated to provide mechanistic insight and suggest a therapeutic role for this compound. Docking studies were also performed for compound **5** on SIRT1 (PDB Id: 4IG9).

## 2. Results and Discussion

### 2.1. Chemistry

**Scheme 1:**
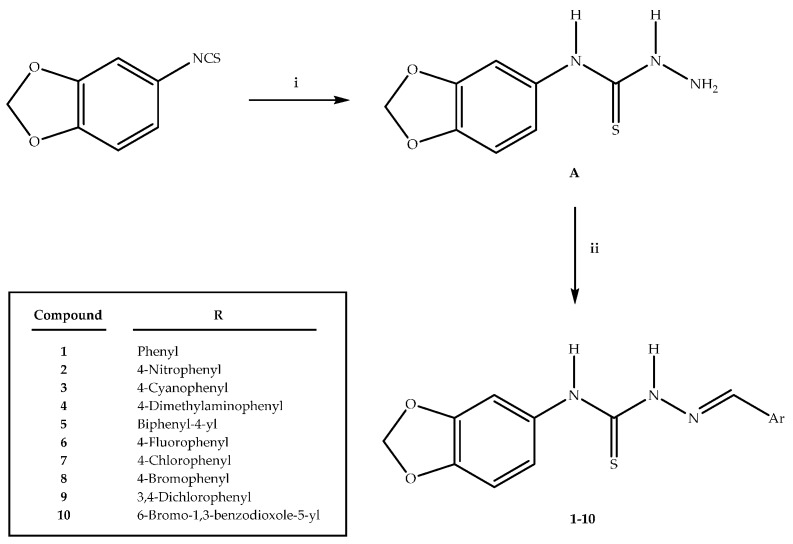
The synthetic route for the preparation of the thiosemicarbazone derivatives **1**–**10**. *Reagents and conditions*: (i) NH_2_NH_2_·H_2_O, ethanol, rt, 4 h; (ii) Ar-CHO, ethanol, reflux, 8 h.

The synthesis of thiosemicarbazone derivatives **1**–**10** was carried out according to the steps depicted in Scheme 1. In the initial step, 4-(1,3-benzodioxole-5-yl)thiosemicarbazide (**A**) was synthesized via the reaction of 1,3-benzodioxole-5-yl isothiocyanate with hydrazine hydrate. The reaction of 4-(1,3-benzodioxole-5-yl)thiosemicarbazide (**A**) with aromatic aldehydes afforded new thiosemicarbazone derivatives **1**–**10**. The IR, ^1^H-NMR, ^13^C-NMR, mass spectral data and elemental analyses were in agreement with the proposed structures of the compounds **1**–**10** (see Supplementary Figures for further details).

### 2.2. Biochemistry

#### 2.2.1. Cytotoxicity

MTT assays were carried out to determine the cytotoxic effects of the compounds on A549 human lung adenocarcinoma and C6 rat glioma cell lines. According to assay results, the tested compounds, except compound **3**, showed more potent inhibitory effects on C6 cells than A549 cells. The most effective cytotoxic agent against A549 cancer cell line was found as compound **5** (IC_50_ = 10.67 ± 1.53 μM) followed by compounds **2**, **3**, **10** and **9** (IC_50_ values were 24.0 ± 3.46, 28.0 ± 1.0, 29.67 ± 5.51 and 51.5 ± 4.95 µM, respectively). Furthermore, the most effective cytotoxic agent against the C6 glioma cell line was found as compound **5** (IC_50_ = 4.33 ± 1.04 μM), followed by compounds **10**, **2**, **9** and **3** (IC_50_ values were 12.33 ± 4.93, 23.33 ± 2.08, 25.33 ± 1.53, and 49.33 ± 1.15 µM, respectively, Table 1).

On the other hand, compounds **1**, **4**, **6**, **7** and **8** showed no significant cytotoxicity at the concentrations used in both cancer cell lines. The cytotoxicity of compounds **1**, **2**, **3**, **4**, **5**, **9** and **10** against NIH/3T3 cells was lower, whereas the cytotoxicity of compounds **6**, **7** and **8** was higher compared to the toxicity on cancer cells (Table 1). These results indicated that the substituents on the benzene ring had considerable influence on the cytotoxic effects of the compounds. Considering the anticancer effects of compound **5**, it can be concluded that biphenyl substituent increased the anticancer activity against A549 human lung adenocarcinoma and C6 rat glioma cell lines. The increased anticancer activity can be attributed to the increased lipophilicity of the compound due to the presence of biphenyl group.

#### 2.2.2. Evaluation of DNA Synthesis Inhibition

The immunostaining procedure was carried out with specific anti-BrdU antibodies in the S-phase of the cell cycle [25]. DNA synthesis inhibitory effects of all compounds were evaluated for 24 h. A549 and C6 cell lines were incubated with three different concentrations of the compounds that were determined according to their IC_50_ values. Cisplatin was used as a positive control. The tested compounds showed DNA synthesis inhibitory activity in a dose-dependent manner.

**Figure 1:**
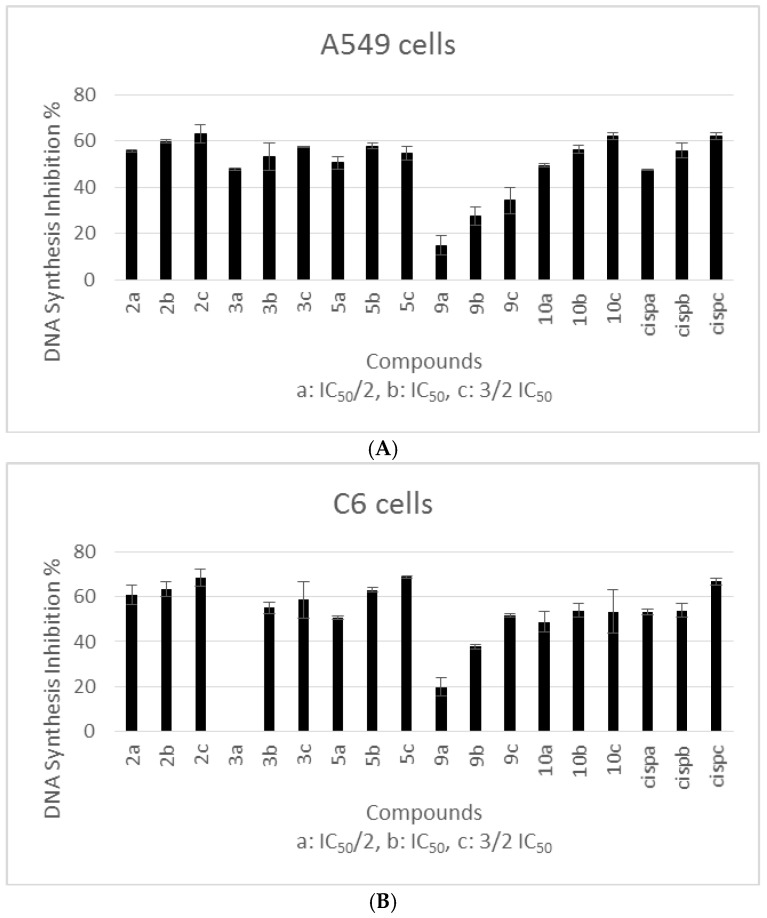
DNA Synthesis inhibitory effects of compounds **2**, **3**, **5**, **9**, **10** and cisplatin on A549 (**A**) and C6 (**B**) cells. Mean percent absorbance of untreated control cells were assumed 0%. Three different concentrations (a: IC_50_/2, b: IC_50_, c: 3/2 IC_50_) of test compounds and cisplatin were given. Data points represent means for two independent experiments ± SD of four independent wells. *p* < 0.05.

The most cytotoxic compounds (compounds **2**, **3**, **5**, **9** and **10**) against A549 and C6 cells were chosen for DNA synthesis inhibition assay. DNA synthesis inhibitory activity of the compounds on A549 and C6 cell lines is presented in Figure 1. These results showed that compounds tested in this assay caused DNA synthesis inhibition. Among these compounds, the most potent inhibitors of DNA synthesis against A549 and C6 cell lines were found as compounds **2**, **5** and **10**. DNA synthesis inhibition caused by the concentration of IC_50_ for each compound tested in A549 cell line revealed the following potency order: Compound **2** > Compound **5** > Compound **10** > Compound **3** > Compound **9**. A significant inhibition of A549 proliferation by cisplatin (positive control) at its IC_50_ value (3.0 ± 0.88 µM) was observed (55.73%). DNA synthesis inhibition percents of compounds **2**, **5**, **10**, **3** and **9** were 59.85%, 57.79%, 56.38%, 53.24%, 27.3%, respectively (Figure 1A). On the other hand, DNA synthesis inhibition caused by the concentration of IC_50_ for each compound tested in C6 cell line revealed the following potency order: Compound **2** > Compound **5** > Compound **3** > Compound **10** > Compound **9**. DNA synthesis inhibition percents of compounds **2**, **5**, **3**, **10** and **9** were 63.34%, 62.98%, 54.99%, 53.96% and 37.71%, respectively, while the inhibition percent of cisplatin was 53.95% at its IC_50_ value (6.83 ± 0.28 µM) (Figure 1B). Consequently, the compounds showed similar antiproliferative activities against both cell lines. Compounds **2**, **5** and **10** were the most cytotoxic agents and exhibited more inhibitory effects on DNA synthesis. In this regard, we investigated possible mechanism of anticancer activity of these compounds.

#### 2.2.3. Evaluation of Flow Cytometric Analyses

##### Apoptosis

**Figure 2:**
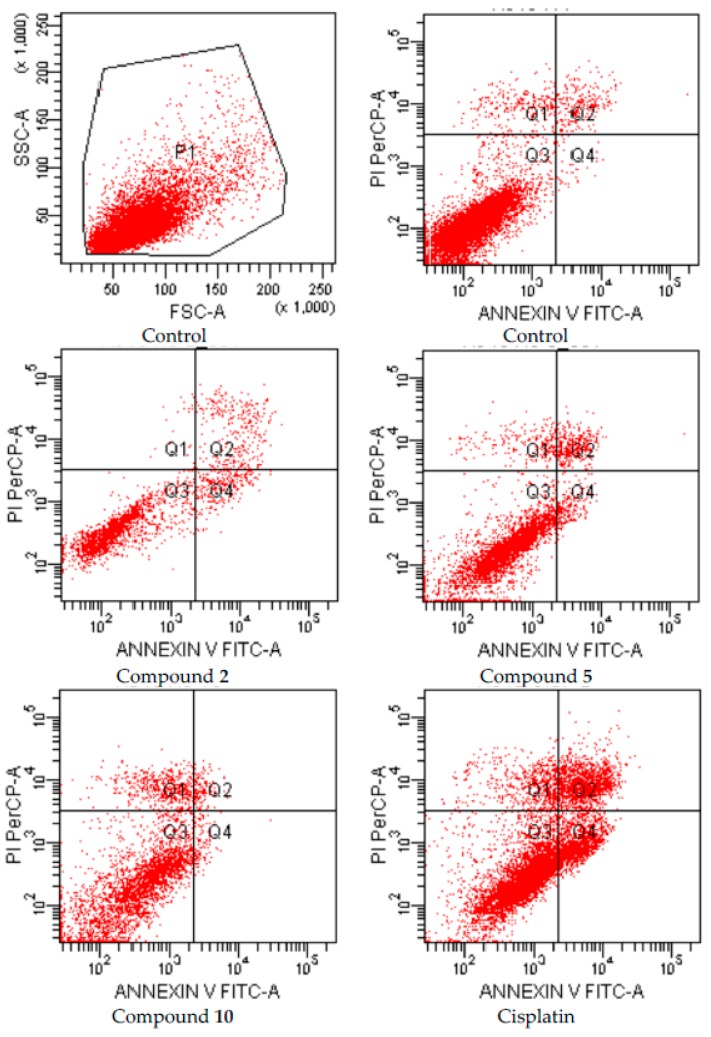
Flow cytometric analysis of A549 cells treated with IC_50_ values of compounds **2**, **5**, **10** and cisplatin. A549 cells were cultured for 24 h in medium with IC_50_ values of the compounds. At least 10,000 cells were analyzed per sample, and quadrant analysis was performed.

**Figure 3:**
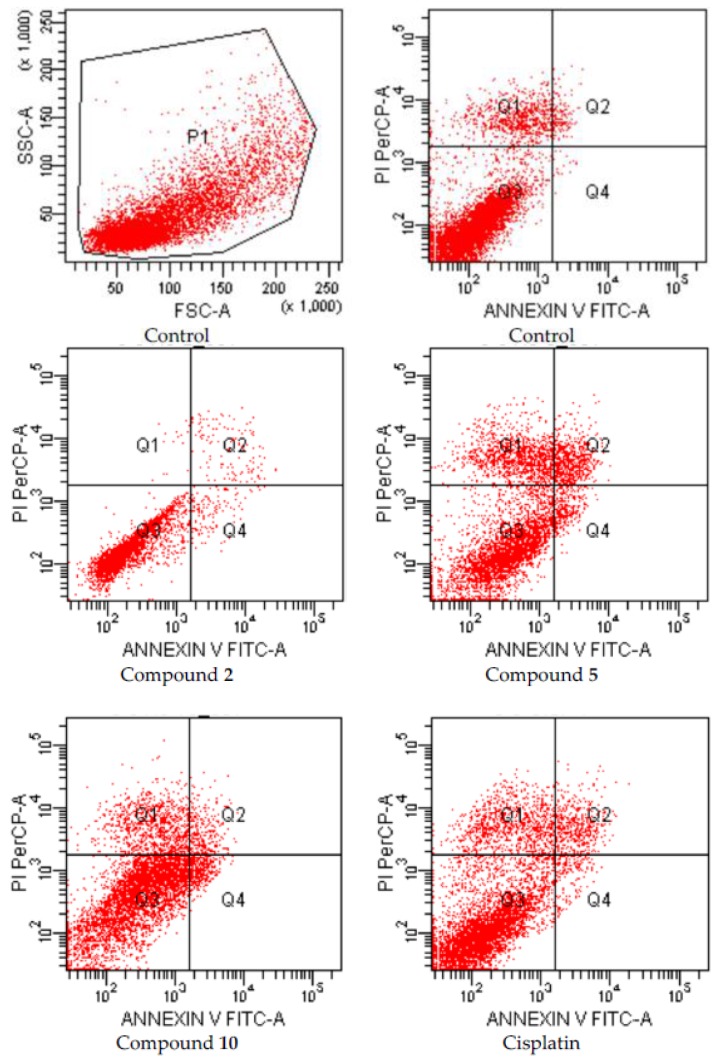
Flow cytometric analysis of C6 cells treated with IC_50_ values of compounds **2**, **5**, **10** and cisplatin. C6 cells were cultured for 24 h in medium with IC_50_ values of the compounds. At least 10,000 cells were analyzed per sample, and quadrant analysis was performed.

After 24 h incubation period, the apoptotic effects of compounds **2**, **5** and **10** which were analyzed for A549 human lung adenocarcinoma and C6 rat glioma cells based on Annexin V-PI binding capacities in flow cytometry are depicted in Figure 2 and Figure 3, respectively. 

Following flow cytometric analyses, early and late apoptotic effects of compounds **2**, **5**, and **10** (for IC_50_ doses) were determined as percentage of 23.7, 12.11 and 6.2, respectively, while early and late apoptotic effects of control cells were determined as percentage of 3.1 on A549 cell line (Figure 2, Table 2).

On the other hand, early and late apoptotic effects of compounds **2**, **5**, and **10** (for IC_50_ doses) were determined as percentage of 5.4, 25.0, and 15.8, respectively, while early and late apoptotic effects of control cells were determined as percentage of 4.5 on C6 cell line (Figure 3, Table 2). According to these findings, compound **5** was the most effective apoptotic compound (25.0%) on C6 cells compared to cisplatin (16.3%). On the other hand, the compounds were not effective as cisplatin (32.4%) on A549 cells.

##### Mitochondrial Membrane Potential (JC-1)

**Figure 4:**
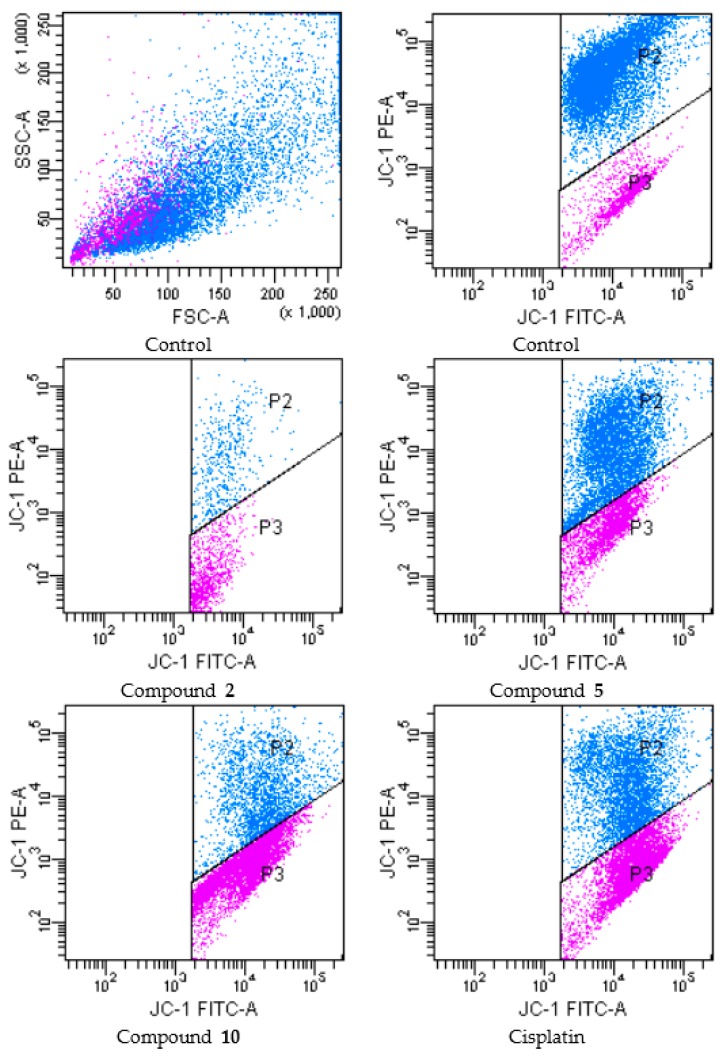
The reduction of the mitochondrial membrane potential in A549 cell line by the compounds. The cells treated or untreated with the IC_50_ doses of the compounds for 24 h were stained with the mitochondrial-selectivite JC-1 dye and analyzed by flow cytometry. P2: mitochondrial membrane polarized cells, P3: mitochondrial membrane depolarized cells.

**Figure 5:**
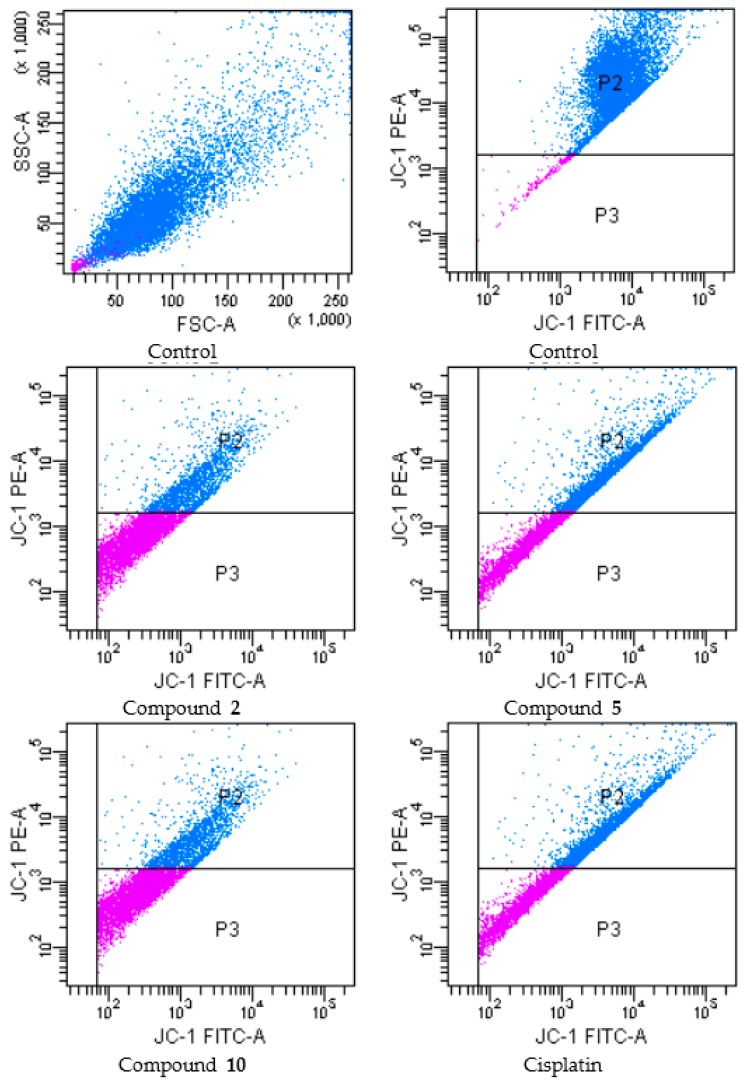
The reduction of the mitochondrial membrane potential in C6 cell line by the compounds. The cells treated or untreated with the IC_50_ doses of the compounds for 24 h were stained with the mitochondrial-selectivite JC-1 dye and analyzed by flow cytometry. P2: mitochondrial membrane polarized cells, P3: mitochondrial membrane depolarized cells.

During apoptosis, several key events occur in mitochondria including the release of cytochrome c, changes in electron transport, and loss of mitochondrial transmembrane potential (ΔΨm). For this reason, ΔΨm is an important parameter of mitochondrial function in apoptosis [26]. In order to investigate effects of compounds **2**, **5** and **10** on mitochondrial membrane potential of A549 and C6 cells, the cells were incubated by IC_50_ concentrations of these compounds for 24 h (Figure 4 and Figure 5 and Table 3, respectively).

Following flow cytometric analyses, mitochondrial membrane polarized cell percentages of compounds **2**, **5**, **10** and cisplatin (for IC_50_ doses) were determined as 4.5, 52.7, 21.9 and 41.4 respectively, while mitochondrial membrane depolarized cell percentages of compounds **2**, **5**, **10** and cisplatin (for IC_50_ doses) were determined as 9.8, 24.6, 57.9 and 54.0 respectively, on A549 cell line. On the other hand, mitochondrial membrane polarized cell percentages of compounds **2**, **5**, **10** and cisplatin (for IC_50_ doses) were determined as 44.8, 49.8, 35.1 and 46.6 respectively, while mitochondrial membrane depolarized cell percentages of compounds **2**, **5**, **10** and cisplatin (for IC_50_ doses) were determined as 21.1, 45.0, 63.7 and 48.4 respectively, on C6 cell line. According to these findings, compound **10** was the most effective agent for depolarization of mitochondrial membrane on both cell lines. Furthermore, compound **5** showed the similar effects on C6 glioma cell line compared to cisplatin.

#### 2.2.4. Evaluation of AChE and BuChE Inhibition

The anticholinesterase effects of all compounds on AChE and BuChE were determined by a modification of Ellman’s spectrophotometric method (Table 4). The tested compounds were more effective against AChE than BuChE. Among these compounds, the most effective AChE inhibitor was found as compound **10** (IC_50_ = 108.0 ± 3.53 µM) followed by compound **9** (IC_50_ = 205.0 ± 5.0 µM) when compared with galantamine (IC_50_ = 1.83 ± 0.21 µM). This outcome indicated that bromo substituted 1,3-benzodioxole ring increased the inhibitory activity against AChE. Compounds **3** and **5** did not show any inhibitory activity against AChE, whereas other derivatives showed weak inhibition on AChE. On the other hand, compounds **7**, **8** and **10** showed weak inhibition on BuChE, whilst other compounds were found to be inactive against BuChE.

Considering the difference between IC_50_ values of compounds **7** and **9**, the introduction of chlorine atom into the 3th position of benzene ring increased anticholinesterase activity against AChE, whereas the introduction of chlorine atom into the 3th position of benzene ring decreased anticholinesterase activity against BuChE.

Compound **5**, the most effective apoptotic derivative in this series against C6 rat glioma cells, did not show any ChE inhibitory activity. On the other hand, compound **10**, the most potent anticholinesterase derivative in this series, did not exhibit significant anticancer activity against A549 and C6 cell lines. In the current work, there is no direct relationship between anticancer activity and cholinesterases.

#### 2.2.5. Evaluation of Sirtuin-1 Activity

**Figure 6:**
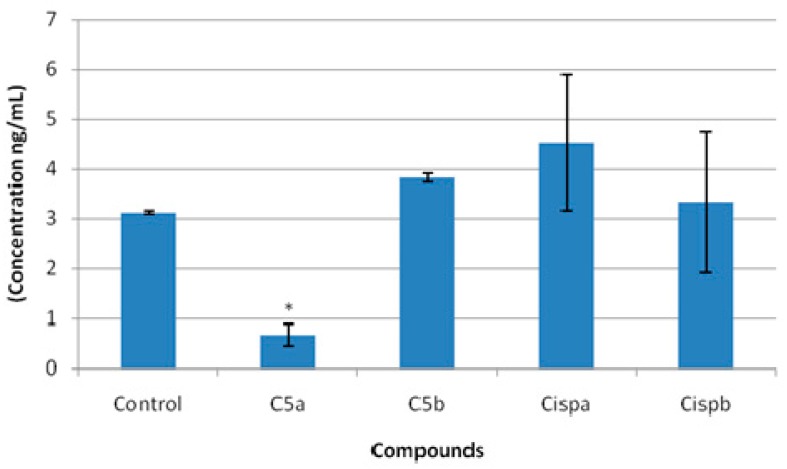
The effects of compound **5** on SIRT1 levels in C6 rat glioma cell line. a: IC_50/2_ concentration; b: IC_50_ concentration; * significance from control group *p* < 0.05.

The effects of compound **5** on sirtuin-1 activity were determined by an ELISA Sirtuin-1 activity kit (Table 5, Figure 6). According to the assay, compound **5** decreased SIRT1 levels in C6 rat glioma cells at IC_50_/2 concentration. However, increasing the concentration of compound **5** caused elevated SIRT1 levels compared to control. These results showed that the effects of compound **5** on SIRT1 levels were dose dependent in C6 glioma cells. In different studies, inhibition of SIRT1 activity leads to elevated p53 acetylation and transactivation resulting in enhanced apoptosis and cytostasis. Hence, inhibition of SIRT1 levels seemed as beneficial for cancer treatment [14].

#### 2.2.6. Docking Studies

**Figure 7:**
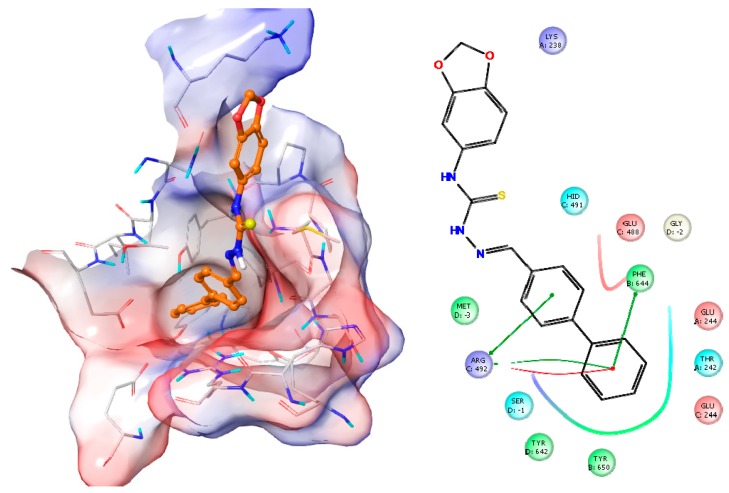
The docking position of compound **5** on SIRT1 and the interactions between compound **5** and SIRT1. (Green line: Pi-pi stacking; Red line: Salt bridge). Surface representation with overlaid electrostatic surface potentials (Red: Negative; Blue: Positive).

Docking studies were also performed for compound **5** on SIRT1 (PDB code: 4IG9). Compound **5** showed good affinity close to active site of the enzyme which was previously defined [12]. In Figure 7, the best docking position of compound **5** on SIRT1 and all interactions between compound **5** and SIRT1 are shown and described.

## 3. Materials and Methods

### 3.1. General Information

All reagents were purchased from commercial suppliers and were used without further purification. Melting points (M.p.) were determined on an Electrothermal 9100 melting point apparatus (Weiss-Gallenkamp, Loughborough, UK) and are uncorrected. IR spectra were recorded on an IRPrestige-21 Fourier Transform Infrared spectrophotometer (Shimadzu, Tokyo, Japan). ^1^H-NMR and ^13^C-NMR spectra were recorded on a Varian Mercury-400 FT-NMR spectrometer (Agilent, Palo Alto, CA, USA). Mass spectra were recorded on an Agilent LC-MSD-Trap-SL Mass spectrometer (Agilent Technologies, Palo Alto, CA, USA). Elemental analyses were performed on a Perkin Elmer EAL 240 elemental analyzer (Perkin-Elmer, Norwalk, CT, USA) and the results were within ±0.4% of the theoretical values.

### 3.2. General Procedures for the Synthesis of the Compounds

#### 3.2.1. 4-(1,3-Benzodioxol-5-yl)thiosemicarbazide (**A**)

A mixture of 1,3-benzodioxol-5-yl isothiocyanate (0.1 mol) and hydrazine hydrate (0.2 mol) in ethanol (30 mL) was stirred at room temperature for 4 h and then filtered. The residue was crystallized from ethanol.

#### 3.2.2. 4-(1,3-Benzodioxol-5-yl)-1-[(aryl)methylene]thiosemicarbazides **1**–**10**

A mixture of 4-(1,3-benzodioxol-5-yl)thiosemicarbazide (**A**) (0.01 mol) and aromatic aldehydes (0.01 mol) was refluxed in ethanol for 8 h, filtered and crystallized from ethanol.

*4-(1,3-Benzodioxole-5-yl)-1-(benzylidene)thiosemicarbazide* (**1**). Yield: 85%. Mp 181.7 °C. IR ν_max_ (cm^−1^): 3317.56 (N-H stretching), 3153.61 (aromatic C-H stretching), 2989.66 (aliphatic C-H stretching), 2883.58 (O-CH_2_ stretching), 1535.34, 1487.12 (C=N, C=C stretching and N-H bending), 1446.61, 1398.39, 1350.17 (C-H bending), 1278.81, 1188.15, 1126.43, 1103.28, 1062.78, 1035.77 (C-N stretching, C=S stretching and aromatic C-H in plane bending), 931.62, 891.11, 854.47, 812.03, 763.81, 750.31, 692.44, 661.58, 624.94 (aromatic C-H out of plane bending and C-S stretching). ^1^H-NMR (400 MHz, DMSO-*d*_6_): 6.04 (s, 2H, O-CH_2_-O), 6.90–6.97 (m, 2H, benzodioxole), 7.19 (s, 1H, benzodioxole), 7.40–7.45 (m, 3H, phenyl), 7.90–7.92 (m, 2H, phenyl), 8.18 (s, 1H, N=CH), 10.02 (s, 1H, NH), 11.82 (s, 1H, NH). ^13^C-NMR (100 MHz, DMSO-*d*_6_): 101.26 (O-C-O), 107.36 (benzodioxole C_4_), 107.97 (benzodioxole C_7_), 119.40 (benzodioxole C_6_), 127.60 (phenyl C_3_ and C_5_), 128.62 (phenyl C_2_ and C_6_), 129.99 (benzodioxole C_5_), 133.10 (phenyl C_4_), 134.04 (phenyl C_1_), 142.78 (benzodioxole C_7a_), 144.87 (N=C), 146.59 (benzodioxole C_3a_), 176.40 (C=S). MS (ESI) (*m/z*): [M + H]^+^ 300. Anal. Calcd. for C_15_H_13_N_3_O_2_S: C, 60.19; H, 4.38; N, 14.04. Found: C, 60.39; H, 4.35; N, 14.30.

*4-(1,3-Benzodioxole-5-yl)-1-(4-nitrobenzylidene)thiosemicarbazide* (**2**). Yield: 95%. Mp 233.1 °C. IR ν_max_ (cm^−1^): 3327.21 (N-H stretching), 3153.61 (aromatic C-H stretching), 2995.45 (aliphatic C-H stretching), 2885.51 (O-CH_2_ stretching), 1585.49, 1334.74 (NO_2_ stretching), 1539.20, 1496.76 (C=N, C=C stretching and N-H bending), 1436.97 (C-H stretching), 1274.95, 1257.59, 1234.44, 1186.22, 1130.29, 1076.28, 1033.85 (C-N stretching, C=S stretching and aromatic C-H in plane bending), 935.48, 898.83, 854.47, 835.18, 808.17, 744.52, 690.52, 599.86 (aromatic C-H out of plane bending and C-S stretching). ^1^H-NMR (400 MHz, DMSO-*d*_6_): 6.06 (s, 2H, O-CH_2_-O), 6.93 (s, 2H, benzodioxole), 7.15 (s, 1H, benzodioxole), 8.17–8.25 (m, 5H, phenyl and N=CH), 10.21 (s, 1H, NH), 12.06 (s, 1H, NH). ^13^C-NMR (100 MHz, DMSO-*d*_6_): 101.32 (O-C-O), 107.41 (benzodioxole C_4_), 108.10 (benzodioxole C_7_), 119.62 (benzodioxole C_6_), 123.70 (phenyl C_3_ and C_5_), 128.40 (phenyl C_2_ and C_6_), 132.92 (benzodioxole C_5_), 140.01 (phenyl C_1_), 140.55 (benzodioxole C_7a_), 145.06 (N=C), 146.66 (benzodioxole C_3a_), 147.63 (phenyl C_4_), 176.83 (C=S). MS (ESI) (*m/z*): [M + H]^+^ 345. Anal. Calcd. for C_15_H_12_N_4_O_4_S: C, 52.32; H, 3.51; N, 16.27. Found: C, 52.22; H, 3.46; N, 16.08.

*4-(1,3-Benzodioxole-5-yl)-1-(4-cyanobenzylidene)thiosemicarbazide* (**3**). Yield: 90%. Mp 224.1 °C. IR ν_max_ (cm^-1^): 3309.85 (N-H stretching), 3142.04 (aromatic C-H stretching), 2981.95 (aliphatic C-H stretching), 2891.30 (O-CH_2_ stretching), 2223.92 (C≡N stretching), 1541.12, 1498.69, 1485.19 (C=N, C=C stretching and N-H bending), 1433.11, 1411.89 (C-H bending), 1298.09, 1253.73, 1215.15, 1180.44, 1138.00, 1082.07, 1031.92 (C-N stretching, C=S stretching and aromatic C-H in plane bending), 923.90, 889.18, 854.47, 862.18, 827.46, 792.74, 731.02, 661.58, 694.37, 599.86 (aromatic C-H out of plane bending and C-S stretching). ^1^H-NMR (400 MHz, DMSO-*d*_6_): 6.06 (s, 2H, O-CH_2_-O), 6.93 (s, 2H, benzodioxole), 7.17 (s, 1H, benzodioxole), 7.87 (d, *J* = 8.4 Hz, 2H, phenyl), 8.11 (d, *J* = 8.8 Hz, 2H, phenyl), 8.19 (s, 1H, N=CH), 10.17 (s, 1H, NH), 12.03 (s, 1H, NH). ^13^C-NMR (100 MHz, DMSO-*d*_6_): 101.32 (O-C-O), 107.40 (benzodioxole C_4_), 108.12 (benzodioxole C_7_), 111.67 (C≡N), 118.79 (benzodioxole C_6_), 119.60 (phenyl C_3_ and C_5_), 128.06 (phenyl C_2_ and C_6_), 132.41 (benzodioxole C_5_), 132.93 (phenyl C_1_), 138.59 (benzodioxole C_7a_), 140.54 (N=C), 145.05 (benzodioxole C_3a_), 146.66 (phenyl C_4_), 176.75 (C=S). MS (ESI) (*m/z*): [M + H]^+^ 325. Anal. Calcd. for C_16_H_12_N_4_O_2_S: C, 59.25; H, 3.73; N, 17.27. Found: C, 60.41; H, 3.75; N, 17.41.

*4-(1,3-Benzodioxole-5-yl)-1-(4-dimethylaminobenzylidene)thiosemicarbazide* (**4**). Yield: 80%. Mp 208.6 °C. IR ν_max_ (cm^−1^): 3282.84 (N-H stretching), 3140.11 (aromatic C-H stretching), 2987.74 (aliphatic C-H stretching), 2881.65 (O-CH_2_ stretching), 1597.06, 1550.77, 1523.76, 1498.69, 1487.12 (C=N, C=C stretching and N-H bending), 1452.40, 1431.18, 1365.60, 1350.17 (C-H bending), 1294.24, 1257.59, 1219.01, 1184.29, 1165.00, 1132.21, 1033.85 (C-N stretching, C=S stretching and aromatic C-H in plane bending), 933.55, 842.89, 813.96, 794.67, 752.24, 709.80, 653.87 (aromatic C-H out of plane bending and C-S stretching). ^1^H-NMR (400 MHz, DMSO-*d*_6_): 2.95 (s, 6H, N(CH_3_)_2_), 6.03 (s, 2H, O-CH_2_-O), 6.71 (d, *J* = 8.8 Hz, 2H, phenyl), 6.89 (d, *J* = 8.4 Hz, 1H, benzodioxole), 6.96 (dd, *J*_1_ = 8.0 Hz and *J*_2_ = 2 Hz, 1H, benzodioxole), 7.22 (d, *J* = 1.6 Hz, 1H, benzodioxole), 7.69 (d, *J* = 8.8 Hz, 2H, phenyl), 8.05 (s, 1H, N=CH), 9.81 (s, 1H, NH), 11.56 (s, 1H, NH). ^13^C-NMR (100 MHz, DMSO-*d*_6_): 39.74 (2CH_3_), 101.19 (O-C-O), 107.30 (benzodioxole C_4_), 107.66 (benzodioxole C_7_), 111.59 (phenyl C_3_ and C_5_), 118.99 (benzodioxole C_6_), 121.18 (phenyl C_1_), 128.98 (phenyl C_2_ and C_6_), 133.27 (benzodioxole C_5_), 143.87 (benzodioxole C_7a_), 144.61 (N=C), 146.51 (benzodioxole C_3a_), 151.47 (phenyl C_4_), 175.27 (C=S). MS (ESI) (*m/z*): [M + H]^+^ 343. Anal. Calcd. for C_17_H_18_N_4_O_2_S: C, 59.63; H, 5.30; N, 16.36. Found: C, 59.60; H, 5.27; N, 16.36.

*4-(1,3-Benzodioxole-5-yl)-1-[(biphenyl-4-yl)methylene]thiosemicarbazide* (**5**). Yield: 90%. Mp 195.7 °C. IR ν_max_ (cm^−1^): 3321.42 (N-H stretching), 3130.47 (aromatic C-H stretching), 2980.02 (aliphatic C-H stretching), 2887.44 (O-CH_2_ stretching), 1600.92, 1531.48, 1502.55, 1485.19 (C=N, C=C stretching and N-H bending), 1440.83, 1354.03 (C-H bending), 1273.02, 1246.02, 1209.37, 1184.29, 1122.57, 1066.64, 1029.99 (C-N stretching, C=S stretching and aromatic C-H in plane bending), 921.97, 840.96, 771.53, 727.16, 702.09, 638.44 (aromatic C-H out of plane bending and C-S stretching). ^1^H-NMR (400 MHz, DMSO-*d*_6_): 6.06 (s, 2H, O-CH_2_-O), 6.93 (d, *J* = 8.4 Hz, 1H, benzodioxole), 7.00 (dd, *J*_1_ = 8.0 Hz, *J*_2_ = 1.6 Hz, 1H, benzodioxole), 7.24 (d, *J* = 1.6 Hz, 1H, benzodioxole), 7.39 (t, *J* = 7.2 Hz, 1H, phenyl), 7.48 (t, *J* = 7.6 Hz, 2H, phenyl), 7.74 (t, *J* = 7.6 Hz, 4H, phenyl), 8.02 (d, *J* = 8.4 Hz, 2H, phenyl), 8.26 (s, 1H, N=CH), 10.11 (s, 1H, NH), 11.92 (s, 1H, NH). ^13^C-NMR (100 MHz) (DMSO-*d*_6_) δ (ppm): 101.68 (CH_2_), 107.79 (CH), 108.49 (CH), 119.93 (CH), 127.13 (2CH), 127.25 (CH), 128.29 (2CH), 129.44 (2CH), 133.53 (CH), 133.61 (CH), 139.79 (2C), 141.85 (CH), 142.71 (C), 145.28 (2C), 146.97 (C), 176.72 (C). MS (ESI) (*m/z*): [M + H]^+^ 376. Anal. Calcd. for C_21_H_17_N_3_O_2_S: C, 67.18; H, 4.56; N, 11.19. Found: C, 67.35; H, 4.59; N, 11.05. 

*4-(1,3-Benzodioxole-5-yl)-1-(4-fluorobenzylidene)thiosemicarbazide* (**6**). Yield: 90%. Mp 196.8 °C. IR ν_max_ (cm^−1^): 3219.19 (N-H stretching), 3118.90 (aromatic C-H stretching), 2981.95 (aliphatic C-H stretching), 2900.94 (O-CH_2_ stretching), 1598.99, 1537.27, 1502.55, 1489.05 (C=N, C=C stretching and N-H bending), 1444.68, 1355.96 (C-H bending), 1228.66, 1211.30, 1188.15, 1157.29, 1072.42, 1037.70 (C-N stretching, C=S stretching and aromatic C-H in plane bending), 937.40, 856.39, 835.18, 810.10, 790.81, 738.74, 711.73, 651.94, 596.00 (aromatic C-H out of plane bending and C-S stretching). ^1^H-NMR (400 MHz, DMSO-*d*_6_): 6.05 (s, 2H, O-CH_2_-O), 6.90-6.95 (m, 2H, benzodioxole), 7.17 (s, 1H, benzodioxole), 7.26 (t, *J* = 8.8 Hz, 2H, phenyl), 7.99 (m, 2H, phenyl), 8.16 (s, 1H, N=CH), 10.04 (s, 1H, NH), 11.81 (s, 1H, NH). ^13^C-NMR (100 MHz, DMSO-*d*_6_): 101.26 (O-C-O), 107.35 (benzodioxole C_4_), 108.03 (benzodioxole C_7_), 115.64 (d, *J* = 21.3 Hz, phenyl C_3_ and C_5_), 119.45 (benzodioxole C_6_), 129.81 (d, *J* = 8.4 Hz, phenyl C_2_ and C_6_), 130.68 (d, *J* = 3.0 Hz, benzodioxole C_5_), 133.09 (phenyl C_1_), 141.57 (benzodioxole C_7a_), 144.89 (N=C), 146.59 (benzodioxole C_3a_), 164.36 (phenyl C_4_), 176.41 (C=S). MS (ESI) (*m/z*): [M + H]^+^ 318. Anal. Calcd. for C_15_H_12_FN_3_O_2_S: C, 56.77; H, 3.81; N, 13.24. Found: C, 56.76; H, 3.42; N, 5.98.

*4-(1,3-Benzodioxole-5-yl)-1-(4-chlorobenzylidene)thiosemicarbazide* (**7**). Yield: 80%. Mp 201.4 °C. IR ν_max_ (cm^−1^): 3344.57 (N-H stretching), 3138.18 (aromatic C-H stretching), 2981.95 (aliphatic C-H stretching), 2895.15 (O-CH_2_ stretching), 1595.13, 1539.20, 1508.33, 1485.19 (C=N, C=C stretching and N-H bending), 1446.61, 1402.25, 1352.10 (C-H bending), 1278.81, 1253.73, 1228.66, 1186.22, 1083.99, 1035.77, 1010.70 (C-N stretching, C=S stretching and aromatic C-H in plane bending), 933.55, 893.04, 846.75, 812.03, 752.24, 659.66, 605.65 (aromatic C-H out of plane bending and C-S stretching). ^1^H-NMR (400 MHz, DMSO-*d*_6_): 6.05 (s, 2H, O-CH_2_-O), 6.90–6.96 (m, 2H, benzodioxole), 7.16 (s, 1H, benzodioxole), 7.48 (d, *J* = 8.4 Hz, 2H, phenyl), 7.95 (d, *J* = 8.8 Hz, 2H, phenyl), 8.14 (s, 1H, N=CH), 10.07 (s, 1H, NH), 11.86 (s, 1H, NH). ^13^C-NMR (100 MHz, DMSO-*d*_6_): 101.26 (O-C-O), 107.35 (benzodioxole C_4_), 108.04 (benzodioxole C_7_), 119.48 (benzodioxole C_6_), 128.65 (phenyl C_3_ and C_5_), 129.21 (phenyl C_2_ and C_6_), 133.02 (benzodioxole C_5_), 133.05 (phenyl C_1_), 134.43 (phenyl C_4_), 141.34 (benzodioxole C_7a_), 144.91 (N=C), 146.59 (benzodioxole C_3a_), 176.48 (C=S). MS (ESI) (*m/z*): [M + H]^+^ 334. Anal. Calcd. for C_15_H_12_ClN_3_O_2_S: C, 53.98; H, 3.62; N, 12.59: C, 53.82; H, 3.50; N, 10.75.

*4-(1,3-Benzodioxole-5-yl)-1-(4-bromobenzylidene)thiosemicarbazide* (**8**). Yield: 82%. Mp 196.3 °C. IR ν_max_ (cm^−1^): 3340.71 (N-H stretching), 3138.18 (aromatic C-H stretching), 2981.95 (aliphatic C-H stretching), 2895.15 (O-CH_2_ stretching), 1589.34, 1537.27, 1485.19 (C=N, C=C stretching and N-H bending), 1446.61, 1398.39, 1354.03 (C-H bending), 1280.73, 1253.73, 1236.37, 1186.22, 1126.43, 1066.64, 1035.77, 1004.91 (C-N stretching, C=S stretching and aromatic C-H in plane bending), 933.55, 893.04, 864.11, 844.82, 812.03, 752.24, 659.66, 601.79 (aromatic C-H out of plane bending and C-S stretching). ^1^H-NMR (400 MHz, DMSO-*d*_6_): 6.05 (s, 2H, O-CH_2_-O), 6.91 (s, 2H, benzodioxole), 7.13 (s, 1H, benzodioxole), 7.61 (d, *J* = 8.8 Hz, 2H, phenyl), 7.87 (d, *J* = 8.8 Hz, 2H, phenyl), 8.11 (s, 1H, N=CH), 10.06 (s, 1H, NH), 11.84 (s, 1H, NH). ^13^C-NMR (100 MHz, DMSO-*d*_6_): 101.24 (O-C-O), 107.34 (benzodioxole C_4_), 108.04 (benzodioxole C_7_), 119.50 (benzodioxole C_6_), 123.22 (phenyl C_4_), 129.46 (phenyl C_3_ and C_5_), 131.55 (phenyl C_2_ and C_6_), 133.03 (benzodioxole C_5_), 133.37 (phenyl C_1_), 141.40 (benzodioxole C_7a_), 144.89 (N=C), 146.56 (benzodioxole C_3a_), 176.47 (C=S). MS (ESI) (*m/z*): [M + H]^+^ 379. Anal. Calcd. for C_15_H_12_BrN_3_O_2_S: C, 47.63; H, 3.20; N, 11.11. Found: C, 47.06; H, 3.37; N, 11.29.

*4-(1,3-Benzodioxole-5-yl)-1-(3,4-dichlorobenzylidene)thiosemicarbazide* (**9**). Yield: 83%. Mp 213.2 °C. IR ν_max_ (cm^−1^): 3228.84 (N-H stretching), 3128.54 (aromatic C-H stretching), 2981.95 (aliphatic C-H stretching), 2889.37 (O-CH_2_ stretching), 1510.26, 1489.05, 1463.97 (C=N, C=C stretching and N-H bending), 1438.90, 1398.39 (C-H bending), 1249.87, 1222.87, 1186.22, 1128.36, 1076.28, 1037.70 (C-N stretching, C=S stretching and aromatic C-H in plane bending), 937.40, 866.04, 815.89, 721.38, 661.58, 601.79 (aromatic C-H out of plane bending and C-S stretching). ^1^H-NMR (400 MHz, DMSO-*d*_6_): 6.06 (s, 2H, O-CH_2_-O), 6.90–6.95 (m, 2H, benzodioxole), 7.13 (d, *J* = 1.6 Hz, 1H, benzodioxole), 7.66 (d, *J* = 8.8 Hz, 1H, phenyl), 7.80 (dd, *J* = 8.4 Hz and *J* = 2 Hz, 1H, phenyl), 8.11 (s, 1H, N=CH), 8.34 (d, *J* = 2 Hz, 1H, phenyl), 10.16 (s, 1H, NH), 11.93 (s, 1H, NH). ^13^C-NMR (100 MHz, DMSO-*d*_6_): 101.28 (O-C-O), 107.37 (benzodioxole C_4_), 108.34 (benzodioxole C_7_), 119.85 (benzodioxole C_6_), 128.03 (phenyl C_5_), 128.32 (phenyl C_3_), 130.69 (phenyl C_6_), 131.80 (phenyl C_2_), 132.03 (benzodioxole C_5_), 132.99 (phenyl C_1_), 134.91 (phenyl C_4_), 139.95 (benzodioxole C_7a_), 145.05 (N=C), 146.62 (benzodioxole C_3a_), 176.73 (C=S). MS (ESI) (*m/z*): [M + H]^+^ 369. Anal. Calcd. for C_15_H_11_Cl_2_N_3_O_2_S: C, 48.93; H, 3.01; N, 11.41. Found: C, 48.48; H, 3.00; N, 11.51.

*4-(1,3-Benzodioxole-5-yl)-1-(6-bromo-1,3-benzodioxole-5-yl)methylene)thiosemicarbazide* (**10**). Yield: 85%. Mp 219.1 °C. IR ν_max_ (cm^−1^): 3224.98 (N-H stretching), 3161.33, 3030.17 (aromatic C-H stretching), 2908.65 (O-CH_2_ stretching), 1531.48, 1504.48, 1469.76 (C=N, C=C stretching and N-H bending), 1444.68, 1419.61 (C-H bending), 1247.94, 1220.94, 1209.37, 1178.51, 1120.64, 1074.35, 1033.85 (C-N stretching, C=S stretching and aromatic C-H in plane bending), 933.55, 879.54, 848.68, 798.53, 746.45, 721.38, 682.80, 632.65, 605.65 (aromatic C-H out of plane bending and C-S stretching). ^1^H-NMR (400 MHz, DMSO-*d*_6_): 6.05 (s, 2H, O-CH_2_-O), 6.13 (s, 2H, O-CH_2_-O), 6.80-6.95 (m, 2H, benzodioxole), 7.11 (d, *J* = 2 Hz, 1H, benzodioxole), 7.24 (s, 1H, benzodioxole), 8.10 (s, 1H, N=CH), 8.45 (s, 1H, benzodioxole), 10.08 (s, 1H, NH), 11.87 (s, 1H, NH). ^13^C-NMR (100 MHz, DMSO-*d*_6_): 101.25 (O-C-O), 102.42 (O-C-O), 106.73 (benzodioxole C_4_), 107.30 (benzodioxole C_4_), 108.26 (benzodioxole C_7_), 112.32 (benzodioxole C_7_), 115.93 (benzodioxole C_6_), 119.71 (benzodioxole C_6_), 126.58 (benzodioxole C_5_), 133.05 (benzodioxole C_5_), 141.25 (benzodioxole C_7a_), 144.92 (N=C), 146.55 (benzodioxole C_3a_), 147.78 (benzodioxole C_3a_), 149.78 (benzodioxole C_7a_), 176.37 (C=S). MS (ESI) (*m/z*): [M + H]^+^ 423. Anal. Calcd. for C_16_H_12_BrN_3_O_4_S: C, 45.51; H, 2.86; N, 9.95. Found: C, 45.84; H, 2.79; N, 9.72.

### 3.3. Biochemistry

#### 3.3.1. Cell Culture and Drug Treatment

C6 Rat glioma and NIH/3T3 mouse embryonic fibroblast cells were incubated in Dulbecco’s Modified Eagle’s Medium (DMEM) (Sigma, Deisenhofen, Germany) supplemented with 10% fetal calf serum (Gibco, Paisley, UK). A549 Human lung adenocarcinoma cells were incubated in 90% RPMI supplemented with 10% fetal bovine serum (Gibco). All media were supplemented with 100 IU/mL penicillin-streptomycin (Gibco) and cells were incubated at 37 °C in a humidified atmosphere of 95% air and 5% CO_2_. Exponentially growing cells were plated at 2 × 10^4^ cells/mL into 96-well microtiter tissue culture plates (Nunc, Roskilde, Denmark) and incubated for 24 h before the addition of the drugs (the optimum cell number for cytotoxicity assays was determined in preliminary experiments). The stock solutions of the compounds were prepared in dimethyl sulfoxide (DMSO; Sigma Aldrich, Poole, UK) and further dilutions were made with fresh culture medium (the concentration of DMSO in the final culture medium was <0.1% which had no effect on the cell viability).

#### 3.3.2. MTT Assay

The level of cellular 3-(4,5-dimethylthiazol-2-yl)-2,5-diphenyltetrazolium bromide (MTT) (Sigma) reduction was quantified as previously described in the literature with small modifications [27,28]. After 24 h of preincubation, the tested compounds and cisplatin (positive control) were added to give final concentration in the range 1.03–167 µM and the cells were incubated for 24 h. At the end of this period, MTT was added to a final concentration of 0.5 mg/mL and the cells were incubated for 4 h at 37 °C. After the medium was removed, the formazan crystals formed by MTT metabolism were solubilized by addition of 200 µL DMSO to each well and absorbance was read at 540 nm with a microtiter plate spectrophotometer (Bio-Tek Plate Reader, Winooski, VT, USA). Every concentration was repeated in three wells. IC_50_ values were defined as the drug concentrations that reduced absorbance to 50% of control values.

#### 3.3.3. Analysis of DNA Synthesis

Analysis of DNA synthesis was measured by a Cell Proliferation ELISA, BrdU (colorimetric) kit (Roche, Mannheim, Germany). This immunostaining procedure is based on measuring the incorporation of bromodeoxyuridine (BrdU) into nuclear DNA in place of thymidine during the S-phase of the cell cycle using specific anti-BrdU antibodies [25]. Hence, such method provides a colorimetric measurement for DNA synthesis inhibition ratio of the carcinogenic cells. Firstly cells were seeded into 96 well flat-bottomed microtiter plates at a density of 2 × 10^3^. The tumor cells were cultured in the presence of various doses of compounds **2**, **3**, **5**, **9**, **10** and cisplatin. Microtiter plates were incubated at 37 °C in a 5% CO_2_/95% air humidified atmosphere for 24 h. Cells were labeled with 10 µL BrdU solution for 2 h and then fixed. Anti-BrdU-POD (100 µL) was added and incubated for 90 min. Finally, wells were washed with BPS and cells were incubated with substrate. Absorbance of the samples was measured with an ELX808-IU Bio-Tek apparatus at 492 nm. All experiments were repeated two times. For each compound dose, dublicate wells were used. 

#### 3.3.4. Flow Cytometric Analyses of Apoptosis

After the cells were incubated with compounds **2**, **5** and **10** at IC_50_ concentrations, phosphatidylserine externalization, which indicates early apoptosis, was measured by Annexin V-PI (BD Pharmingen, San Jose, CA, USA) on a BD FACSCalibur^TM^ flow cytometer for 24 h. Annexin V staining protocol was applied according to the manufacturer’s instructions (BD Pharmingen). The cells were then briefly washed with cold phosphate buffer saline (PBS) and suspended in a binding buffer at a concentration of 1 × 10^6^ cells/mL. Then, 100 µL of this solution containing 1 × 10^5^ cells was transferred to a 5 mL test tube. After 5 µL of Annexin-V and PI was added, the cells were incubated for 15 min at room temperature in the dark. Then 400 µL of 1x Binding Buffer was added to each tube and the cells were processed for data acquisition, and analyzed on a Becton–Dickinson FACS Aria using FACSDIVA Version 6.1.1. Software (BD Biosciences, San Jose, CA, USA).

#### 3.3.5. Analysis of Mitochondrial Membrane Potential (JC-1) by Flow Cytometry

The cells were seeded in six-well plates at a density of 10^5^ cells/mL, and the IC_50_ dose of compounds **2**, **5** and **10** was added to cells. The cells were incubated in 5% CO_2_ air-conditioned atmosphere at 37 °C. After 48 h of incubation, the cells were trypsinized, washed with PBS, and centrifuged at 400× *g* for 5 min. 5,5′,6,6′-Tetrachloro-1,1′,3,3′-tetraethylbenzimidazolylcarbocyanine iodide (JC-1) dye solution (1× assay buffer + JC-1 stock solution) was added to the cells. The stock solution was prepared by dissolving DMSO. Then the samples were incubated at a temperature of 37 °C for 10–15 min. After incubation, the cells were washed twice with an assay buffer and analyzed by BD FACS Aria Cell Sorter Software version 6.1.1 flow cytometry (BD Biosciences). The cells showing mitochondrial membrane potential disruption were determined as a percentage of all cells.

#### 3.3.6. AChE and BuChE Inhibitory Activity

AChE and BuChE inhibitory effects of the compounds were determined by Ellman’s method with minor modifications (Electric eel acetylcholinesterase enzyme was used instead of bovine acetylcholinesterase enzyme and buffer was added 2.4 mL instead of 3 mL) [29]. The compounds were dissolved in DMSO and tested at final concentration range 5–80 μg/mL. 20 μL of enzyme (AChE or BuChE, 1 U/mL), 10 μL sample added to 2.4 mL buffer, the mixture was incubated at 37 °C for 15 min. After 15 min incubation, 50 μL of 0.01 M 5,5′-dithiobis(2-nitrobenzoic acid) (DTNB) and 20 μL of 75 mM acetylthiocholine iodide (ATCI) or 25 mM butyrylthiocholine iodide (BTCI) were added, and the final mixture was incubated at room temperature for 30 min. Blank was prepared using 10 μL of DMSO instead of the test sample, with all other procedures similar to those used in the case of the sample mixture. Absorbances were measured at 412 nm and 37 °C using polystyrene cuvettes with spectrophotometer (UV-1700, Shimadzu). Experiment was done in triplicate. Galantamine was used as a positive control. Data are expressed as mean ± standard deviation (SD). The inhibition (percent) of AChE or BuChE was calculated using the following equation:

I (%) = 100 − (OD_sample_/OD_control_) × 100

#### 3.3.7. Sirtuin-1 Activity Detection by ELISA

C6 glioma cells were administrated by IC_50_ or IC_50_/2 concentrations of compound **5** and cisplatin for 24 h. Cells were detached with trysin and then collected by centrifugation. After cells were washed by 1× PBS for three times, cell lysates were prepared by frozen cells at ≤−20 °C. Freeze/thaw cycles were repeated for three times. Then cells were centrifuged at 1500× *g* for 10 min at 2–8 °C to remove cellular debris. Rat Sirtuin-1 activity protocol was applied according to the manufacturer’s instructions (USCN, Life Science Inc., Wuhan, China). Briefly, all standards, reagents and samples were prepared. 100 µL standard or sample were added to each well and incubated for 2 h at 37 °C. 100 µL detection reagent A added and incubated 1 h at 37 °C. Then solutions were aspirated and wells were washed 3 times. Then 100 µL detection reagent B was added and incubated for 30 min at 37 °C. Solutions were aspirated and washed 5 times. Then 90 µL substrate solution added and incubated 15–25 min at 37 °C. After 50 µL stop solution was added, plate was read at 450 nm with a microtiter plate spectrophotometer (Bio-Tek Plate Reader). Every concentration was repeated in double wells. The results were given as ng/mL.

#### 3.3.8. Statistical Analyses

Statistical Package for the Social Sciences (SPSS, Chicago, IL, USA) for Windows 15.0 was used for statistical analysis. Data was expressed as Mean ± SD. Comparisons were performed by one way ANOVA test for normally distributed continuous variables and post hoc analyses of group differences were expressed by the Tukey test. Also, Date were compared between two groups using Student’s *t-*test. The *p* < 0.05 was considered as statistically significant in this study.

#### 3.3.9. Docking Studies

Compound **5** was docked to the active site of 4IG9. Ligand was set to the physiological pH (pH = 7.4) at the protonation step and crystal structure of SIRT1 was retrieved from Protein Data Bank server, (PDB code: 4IG9). The structure of compound **5** was submitted in protein preparation module of Schrodinger’s Maestro molecular modeling package. In molecular docking simulations: Glide/XP docking protocols were applied for the prediction of topologies of compound **5** at the active site of target structure. The formation of active site in the enzyme was determined from previous studies [12].

## 4. Conclusions

In the present paper, new benzodioxole-based thiosemicarbazone derivatives were synthesized and evaluated for their cytotoxicity against on A549 human lung adenocarcinoma, C6 rat glioma and NIH/3T3 mouse embryonic fibroblast cell lines. In order to examine the correlation between anticancer activity with cholinesterases, each derivative was investigated for its ability to inhibit AChE and BuChE. The most effective compounds were also investigated for their effects on DNA synthesis, apoptosis and mitochondrial membrane potential.

Among these compounds, compound **5** can be considered as the most promising anticancer agent against C6 (IC_50_ = 4.33 ± 1.04 μM) and A549 (IC_50_ = 10.67 ± 1.53 μM) cells and low toxicity to NIH/3T3 (IC_50_ = 21.33 ± 5.77 μM) cells. Compound **5** showed its anticancer activity against C6 cell line via the induction of apoptotic pathway, but this effect of compound **5** was independent of ChE inhibitory activity. The effects of compound **5** on SIRT1 activity were evaluated for underlying mechanism of apoptosis. The results indicated that compound **5** decreased SIRT1 levels in C6 rat glioma cells at IC_50_/2 concentration, whereas compound **5** caused elevated SIRT1 levels compared to control at increased concentrations. This outcome pointed out that the effects of compound **5** on SIRT1 levels were dose dependent in C6 glioma cells. Docking studies were also carried out for compound **5** on SIRT1 to enlighten the exact position and interactions of this compound on the active site of enzyme.

## Figures and Tables

**Table 1:** IC_50_ values of the compounds against A549, C6 and NIH/3T3 cells for 24 h.

Compound	IC_50_ (µM)		
	A549 Cell Line	C6 Cell Line	NIH/3T3 Cell Line
**1**	>167.0	>167.0	>167.0
**2**	24.0 ± 3.46	23.33 ± 2.08	>145.2
**3**	28.0 ± 1.0	49.33 ± 1.15	>154.1
**4**	>146.0	>146.0	>146.0
**5**	10.67 ± 1.53	4.33 ± 1.04	21.33 ± 5.77
**6**	>157.6	>157.6	2.87 ± 0.05
**7**	>149.7	>149.7	9.50 ± 2.50
**8**	>132.2	>132.2	9.16 ± 2.47
**9**	51.5 ± 4.95	25.33 ± 1.53	>135.8
**10**	29.67 ± 5.51	12.33 ± 4.93	38.33 ± 2.08
Cisplatin	3.0 ± 0.88	6.83 ± 0.28	ND

ND: Not determined.

**Table 2:** The percents of typical quadrant analysis of annexin V-FITC/propidium iodide flow cytometry of A549 and C6 cells treated with the compounds.

Groups	Early and Late Apoptotic Cells %		Viability %	
	C6 Cells	A549 Cells	C6 Cells	A549 Cells
Control (untreated)	4.5	3.1	83.9	94.3
Compound **2** treated cells	5.4	23.7	94.5	75.2
Compound **5** treated cells	25.0	12.11	60.2	81.8
Compound **10** treated cells	15.8	6.2	69.6	81.2
Cisplatin treated cells	16.3	32.4	69.0	60.5

C6 and A549 cells were cultured for 24 h in medium with IC_50_ values of the compounds. At least 10,000 cells were analyzed per sample, and quadrant analysis was performed.

**Table 3:** The effects of the compounds on mitochondrial membrane potential of A549 and C6 cells for 24 h.

Groups	P2 (%)		P3 (%)	
	C6 Cells	A549 Cells	C6 Cells	A549 Cells
Control (untreated)	98.3	82.6	1.7	13.1
Compound **2** treated cells	44.8	4.5	21.1	9.8
Compound **5** treated cells	49.8	52.7	45.0	24.6
Compound **10** treated cells	35.1	21.9	63.7	57.9
Cisplatin treated cells	46.6	41.4	48.4	54.0

**Table 4:** The anticholinesterase activity of the compounds.

Compound	IC_50_ (µM)	
	AChE	BuChE
**1**	>267.25	---
**2**	>232.32	---
**3**	---	---
**4**	>233.68	---
**5**	---	---
**6**	>252.10	---
**7**	>239.67	>239.67
**8**	>211.51	>211.51
**9**	205.0 ± 5.0	---
**10**	108.0 ± 3.53	>189.46
Galantamine	1.83 ± 0.21	18.17 ± 0.76

---: No inhibition.

**Table 5:** The effects of compound **5** on SIRT1 Activity.

Compound	SIRT1 Concentration (ng/mL)
Control	3.12 ± 0.03
Compound **5** a	0.67 ± 0.22
Compound **5** b	3.82 ± 0.08
Cisplatin a	4.53 ± 1.37
Cisplatin b	3.32 ± 1.41

a: IC_50_/2 concentration; b: IC_50_ concentration.

## Supplementary Materials

The following are available online at http://www.mdpi.com/1420-3049/21/11/1598/s1.

## References

[1] Silverstein A., Silverstein V., Silverstein Nunn L. (2006). Cancer, Conquering a Deadly Disease.

[2] Ruddon R.W. (2007). Cancer Biology.

[3] Almeida C.A., Barry S.A. (2010). Cancer: Basic Science and Clinical Aspects.

[4] Giamas G., Man Y.L., Hirner H., Bischof J., Kramer K., Khan K., Ahmed S.S., Stebbing J., Knippschild U. (2010). Kinases as targets in the treatment of solid tumors. Cell Signal.

[5] Von Mehren M., Widmer N. (2011). Correlations between imatinib pharmacokinetics, pharmacodynamics, adherence, and clinical response in advanced metastatic gastrointestinal stromal tumor (GIST): An emerging role for drug blood level testing?. Cancer Treat. Rev..

[6] Sestak V., Stariat J., Cermanova J., Potuckova E., Chladek J., Roh J., Bures J., Jansova H., Prusa P., Sterba M. (2015). Novel and potent anti-tumor and anti-metastatic di-2-pyridylketone thiosemicarbazones demonstrate marked differences in pharmacology between the first and second generation lead agents. Oncotarget.

[7] Massoulié J., Perrier N., Noureddine H., Liang D., Bon S. (2008). Old and new questions about cholinesterases. Chem. Biol. Interact..

[8] Pérez-Aguilar B., Vidal C.J., Palomec G., García-Dolores F., Gutiérrez-Ruiz M.C., Bucio L., Gómez-Olivares J.L., Gómez-Quiroz L.E. (2015). Acetylcholinesterase is associated with a decrease in cell proliferation of hepatocellular carcinoma cells. Biochim. Biophys. Acta.

[9] Xi H.-J., Wu R.-P., Liu J.-J., Zhang L.-J., Li Z.-S. (2015). Role of acetylcholinesterase in lung cancer. Thorac. Cancer.

[10] Firestein R., Blander G., Michan S., Oberdoerffer P., Ogino S., Campbell J., Bhimavarapu A., Luikenhuis S., de Cabo R., Fuchs C. (2008). The SIRT1 deacetylase suppresses intestinal tumorigenesis and colon cancer growth. PLoS ONE.

[11] Finkel T., Deng C.X., Mostoslavsky R. (2009). Recent progress in the biology and physiology of sirtuins. Nature.

[12] Davenport A.M., Huber F.M., Hoelz A. (2014). Structural and functional analysis of human SIRT1. J. Mol. Biol..

[13] Feng H., Guo P., Wang J., Xu J., Xie C., Gao F. (2016). Expression of Leptin and Sirtuin-1 is associated with poor prognosis in patients with osteosarcoma. Pathol. Res. Pract..

[14] Lin Z., Fang D. (2013). The Roles of SIRT1 in Cancer. Genes Cancer.

[15] Kalinowski D.S., Quach P., Richardson D.R. (2009). Thiosemicarbazones: The new wave in cancer treatment. Future Med. Chem..

[16] Chapman T.R., Kinsella T.J. (2012). Ribonucleotide reductase inhibitors: A new look at an old target for radiosensitization. Front. Oncol..

[17] Moorthy N.S., Cerqueira N.M., Ramos M.J., Fernandes P.A. (2013). Aryl- and heteroaryl-thiosemicarbazone derivatives and their metal complexes: A pharmacological template. Recent Pat. Anticancer Drug Discov..

[18] Yu Y., Kalinowski D.S., Kovacevic Z., Siafakas A.R., Jansson P.J., Stefani C., Lovejoy D.B., Sharpe P.C., Bernhardt P.V., Richardson D.R. (2009). Thiosemicarbazones from the old to new: İron chelators that are more than just ribonucleotide reductase inhibitors. J. Med. Chem..

[19] Stefani C., Al-Eisawi Z., Jansson P.J., Kalinowski D.S., Richardson D.R. (2015). Identification of differential anti-neoplastic activity of copper bis(thiosemicarbazones) that is mediated by intracellular reactive oxygen species generation and lysosomal membrane permeabilization. J. Inorg. Biochem..

[20] Jagadeesh M., Rashmi H.K., Subba Rao Y., Sreenath Reddy A., Prathima B., Uma Maheswari Devi P., Reddy A.V. (2013). Synthesis and spectroscopic characterization of 3,4-difluoroacetophenone-thiosemicarbazone and its palladium(II) complex: Evaluation of antimicrobial and antitumour activity. Spectrochim. Acta A Mol. Biomol. Spectrosc..

[21] Taşdemir D., Karaküçük-İyidoğan A., Ulaşli M., Taşkin-Tok T., Oruç-Emre E.E., Bayram H. (2015). Synthesis, molecular modeling, and biological evaluation of novel chiral thiosemicarbazone derivatives as potent anticancer agents. Chirality.

[22] Bacher F., Dömötör O., Chugunova A., Nagy N.V., Filipović L., Radulović S., Enyedy É.A., Arion V.B. (2015). Strong effect of copper(II) coordination on antiproliferative activity of thiosemicarbazone-piperazine and thiosemicarbazone-morpholine hybrids. Dalton Trans..

[23] Lin Z.P., Ratner E.S., Whicker M.E., Lee Y., Sartorelli A.C. (2014). Triapine disrupts CtIP-mediated homologous recombination repair and sensitizes ovarian cancer cells to PARP and topoisomerase inhibitors. Mol. Cancer Res..

[24] Wang H.H., Qiu K.M., Cui H.E., Yang Y.S., Yin-Luo, Xing M., Qiu X.Y., Bai L.F., Zhu H.L. (2013). Synthesis, molecular docking and evaluation of thiazolyl-pyrazoline derivatives containing benzodioxole as potential anticancer agents. Bioorg. Med. Chem..

[25] Malikova J., Swaczynova J., Kolar Z., Strnad M. (2008). Anticancer and antiproliferative activity of natural brassinosteroids. Phytochemistry.

[26] Green D.R., Reed J.C. (1998). Mitochondria and apoptosis. Science.

[27] Mosmann T. (1983). Rapid colorimetric assay for cellular growth and survival: Application to proliferation and cytotoxicity assays. J. Immunol. Methods.

[28] Keiser K., Johnson C.C., Tipton D.A. (2000). Cytotoxicity of mineral trioxide aggregate using human periodontal ligament fibroblasts. J. Endod..

[29] Ellman G.L., Courtney K.D., Anders V., Featherstone R.M. (1961). A new and rapid colorimetric determination of acetylcholinesterase activity. Biochem. Pharmacol..

