# Supplementary material for: Synthesis and Evaluation of New Benzodioxole- Based Thiosemicarbazone Derivatives as Potential Antitumor Agents
Source: Molecules. 2016 Nov 22;21(11):1598. doi: 10.3390/molecules21111598 (PMC6273715; doi:10.3390/molecules21111598)
Supplement: Supplementary file 1 [file molecules-21-01598-s001.pdf]

Supplementary Materials: Synthesis and Evaluation of New Benzodioxole-Based Thiosemicarbazone Derivatives as Potential Antitumor Agents

Mehlika Dilek Altıntop, Halide Edip Temel, Belgin Sever, Gülşen Akalın Çiftçi and Zafer Asım Kaplancıklı

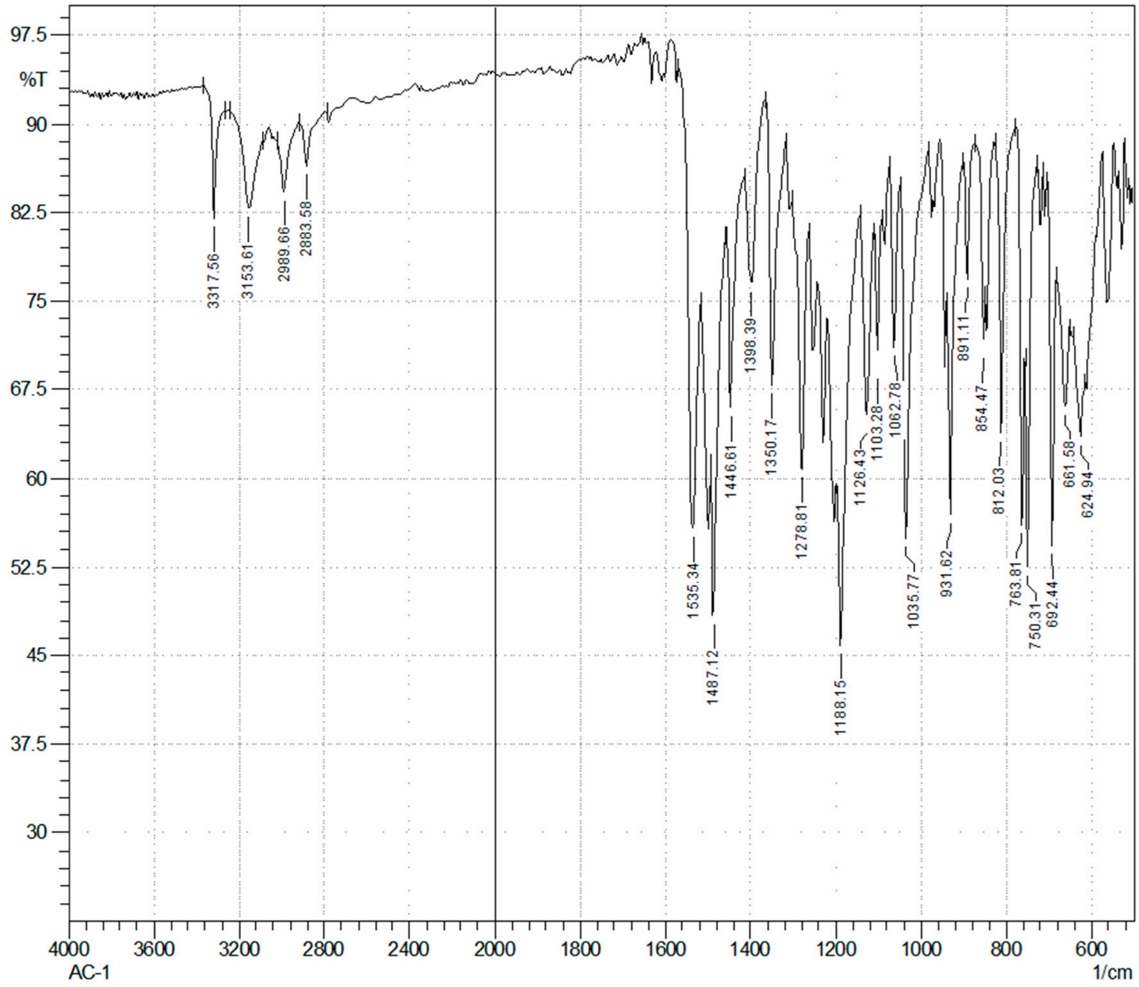

Figure S1. IR Spectrum of Compound **1**.

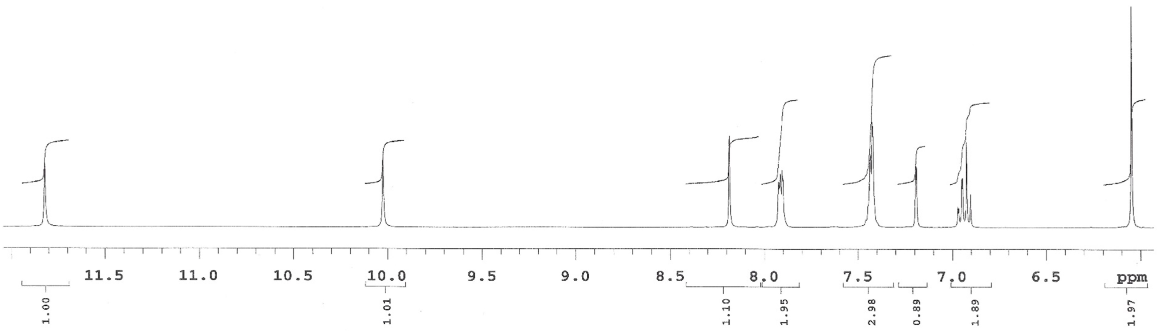

Figure S2. ^{1}H-NMR Spectrum of Compound **1** (6.5–11.5 ppm).

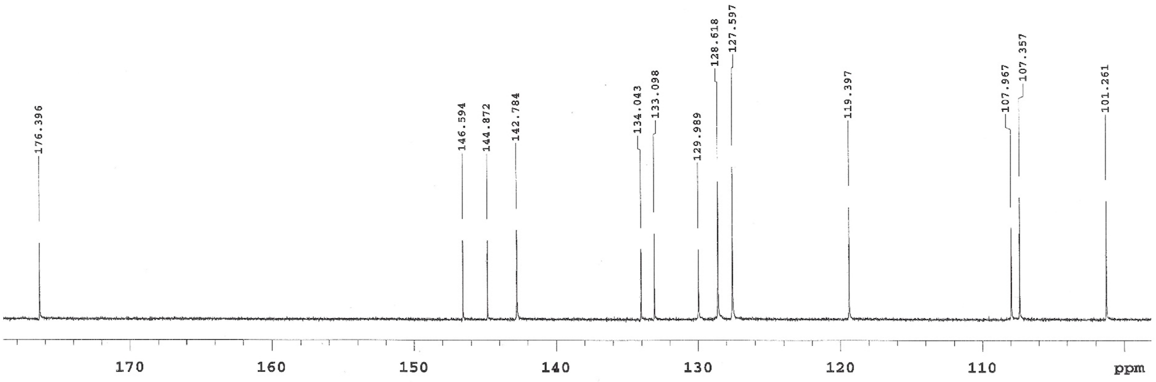

Figure S3. ^{13}C-NMR Spectrum of Compound **1** (101–176 ppm).

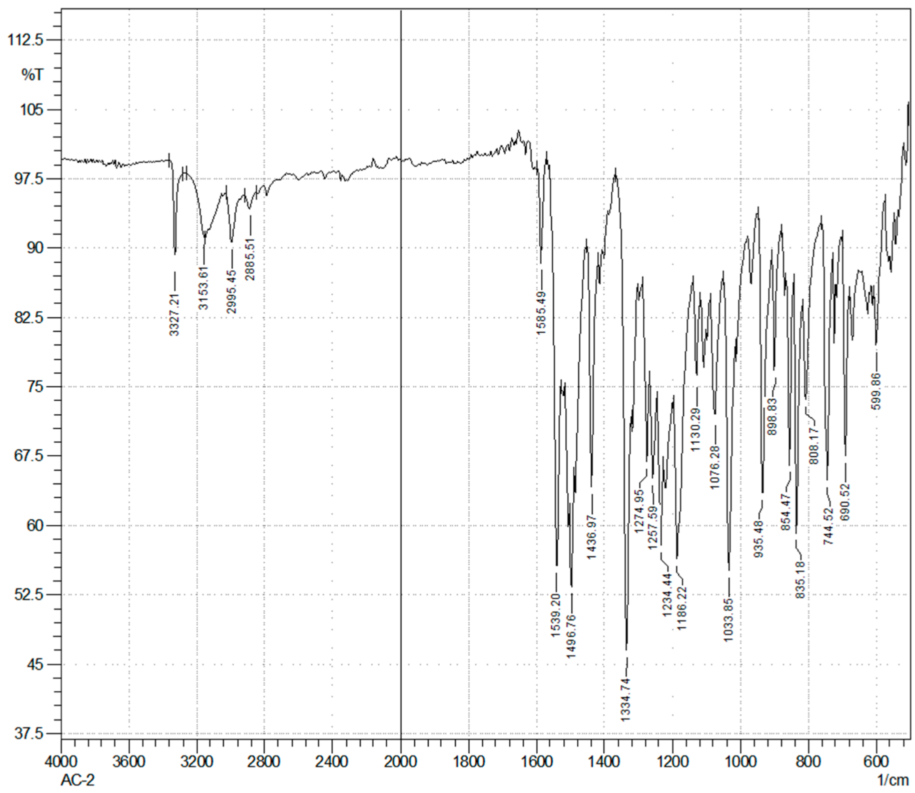

Figure S4. IR Spectrum of Compound **2**.

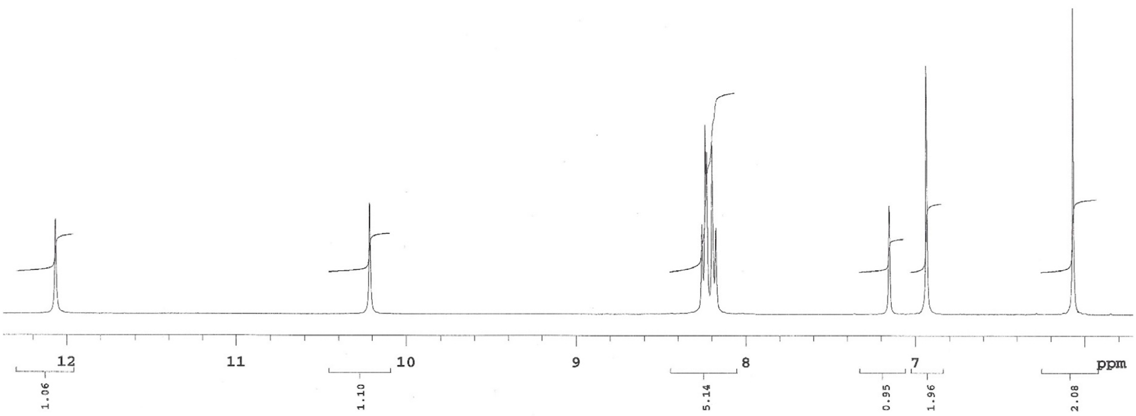

Figure S5. ^{1}H-NMR Spectrum of Compound **2** (35–44 ppm).

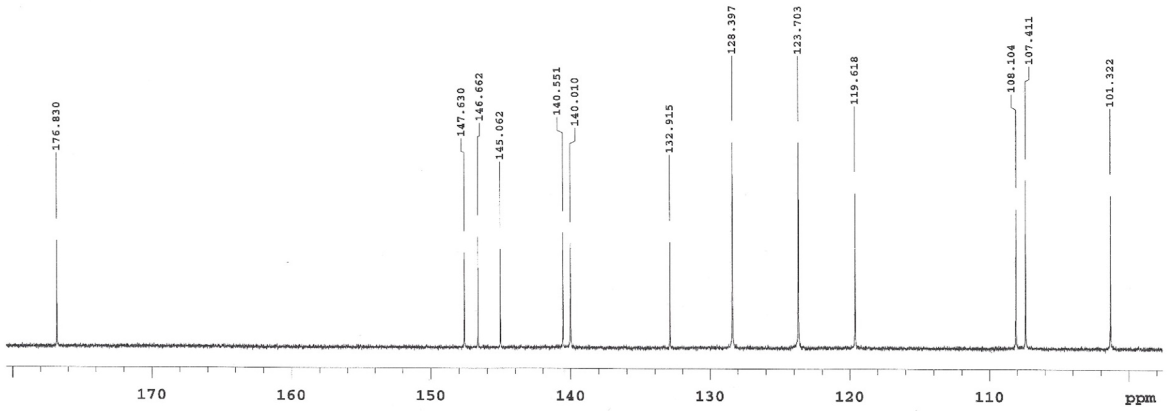

Figure S6. ^{13}C-NMR Spectrum of Compound **2** (110–170 ppm).

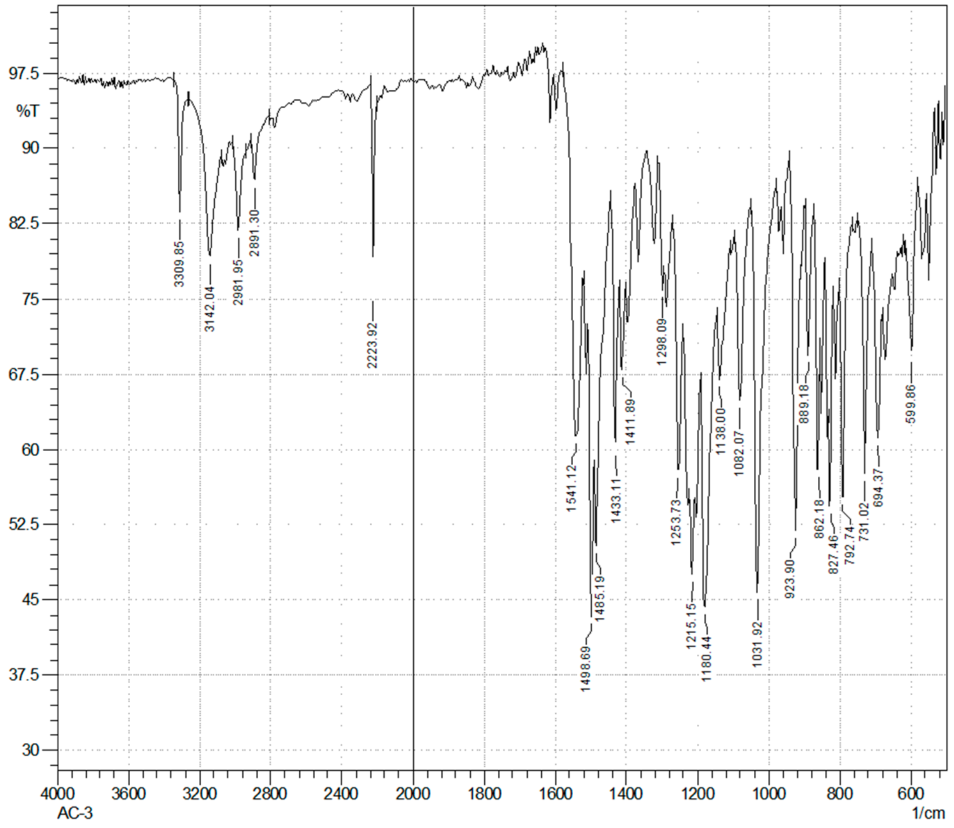

Figure S7. IR Spectrum of Compound **3.**

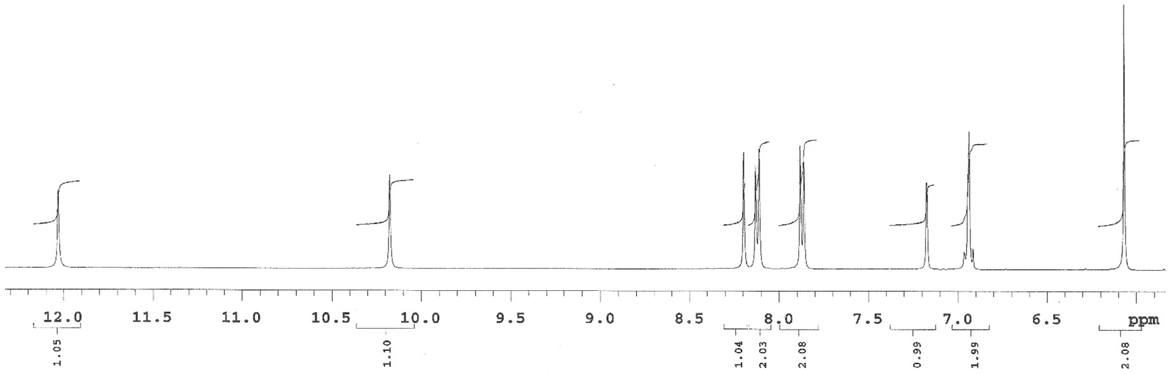

Figure S8. ^{1}H-NMR Spectrum of Compound **3** (6.5–12 ppm).

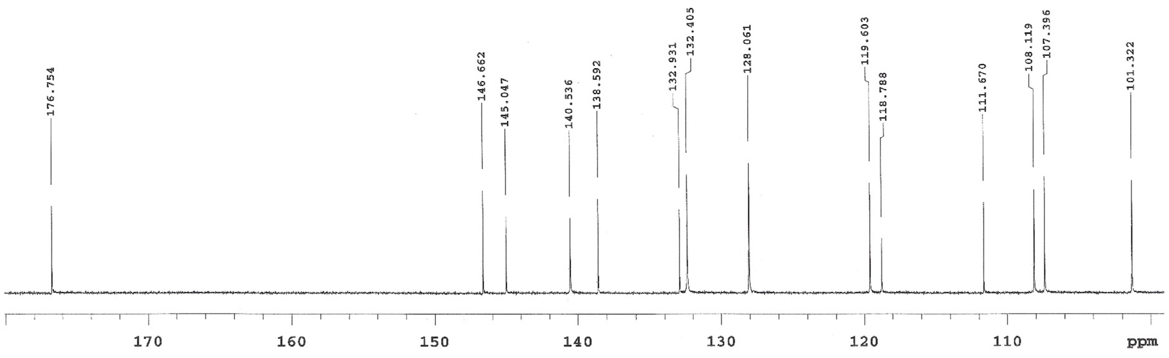

Figure S9. ^{13}C-NMR Spectrum of Compound **3** (101–176 ppm).

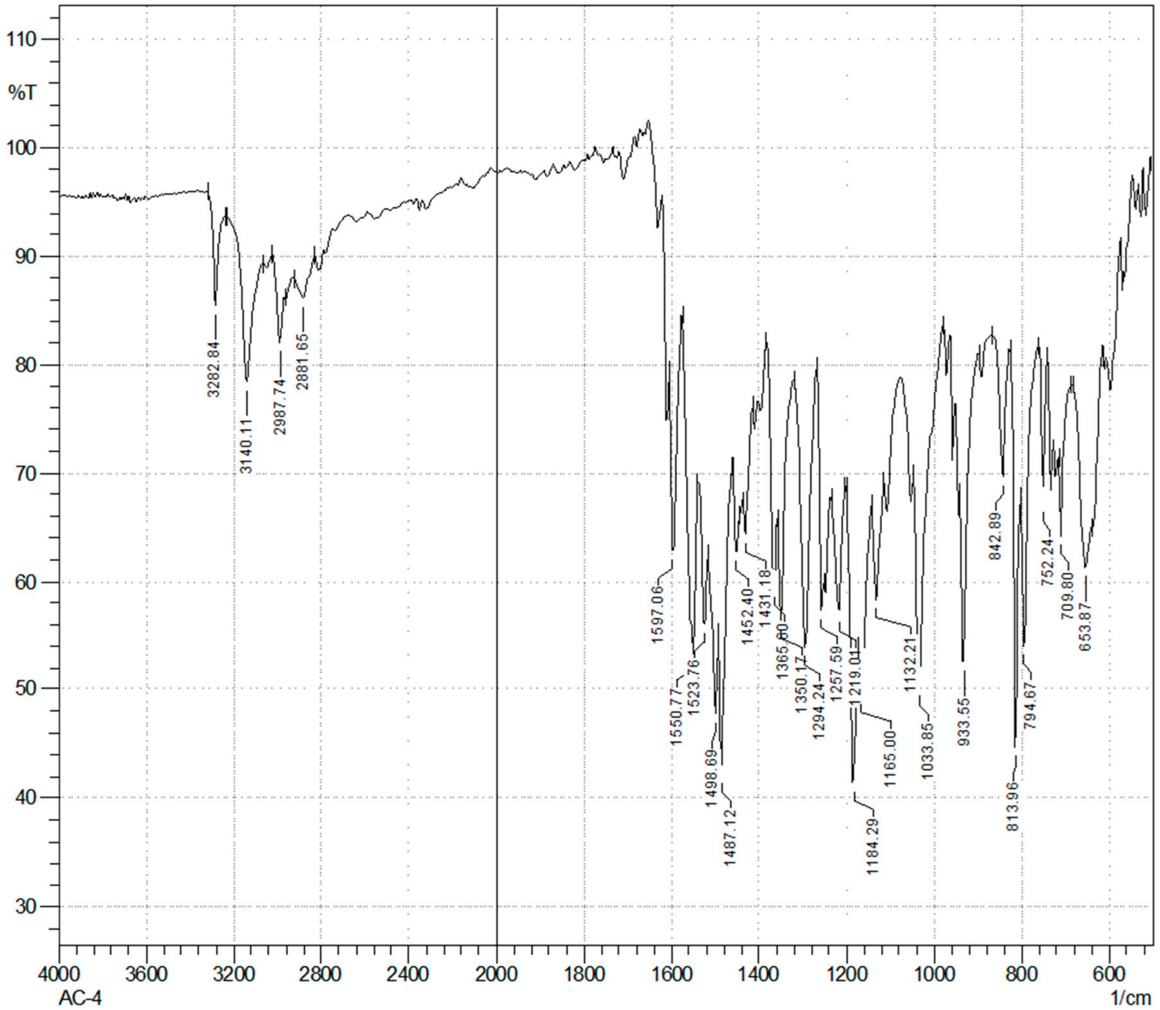

Figure S10. IR Spectrum of Compound **4**.

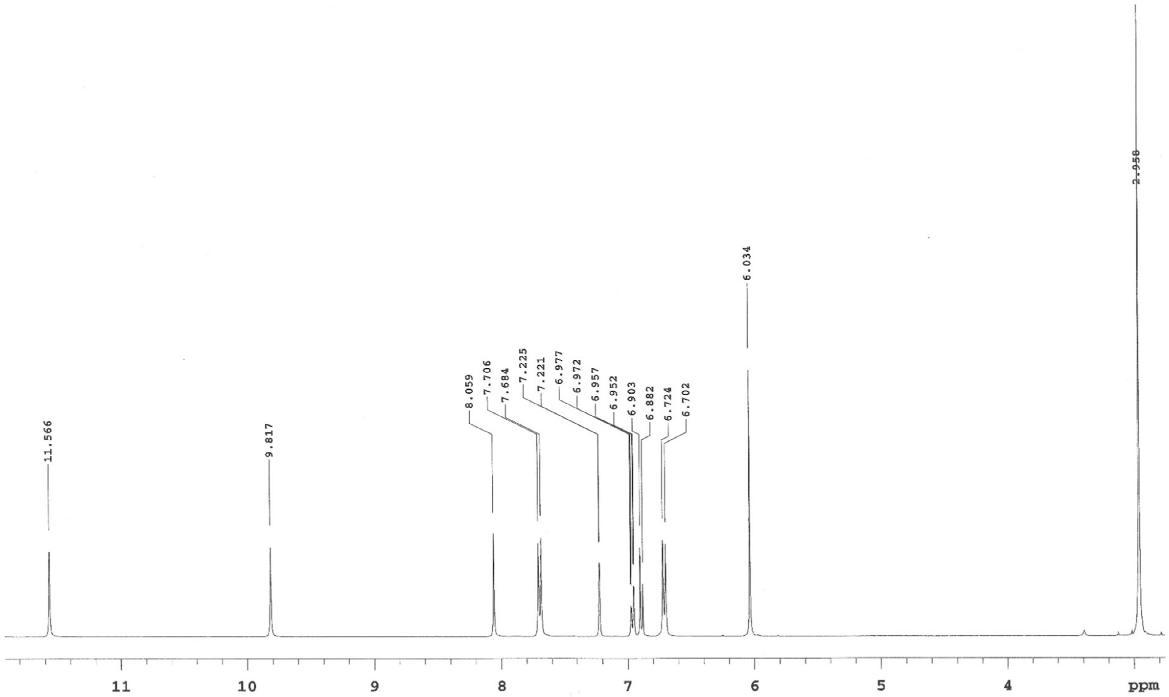

Figure S11. ^{1}H-NMR Spectrum of Compound **4** (4–11 ppm).

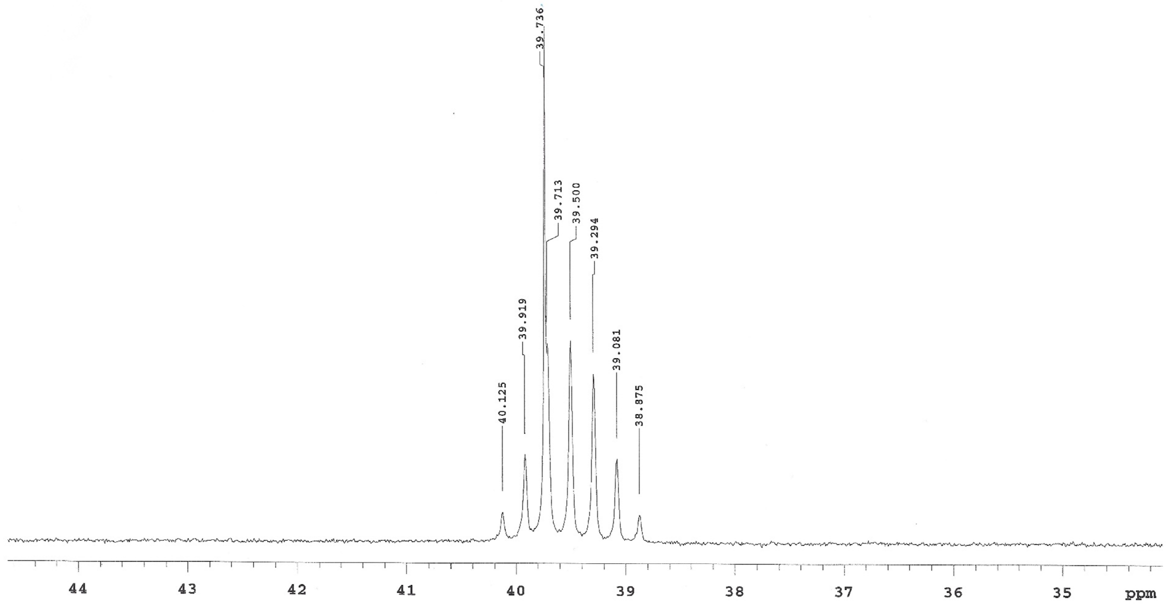

Figure S12. ^{13}C-NMR Spectrum of Compound **4** (35–44 ppm).

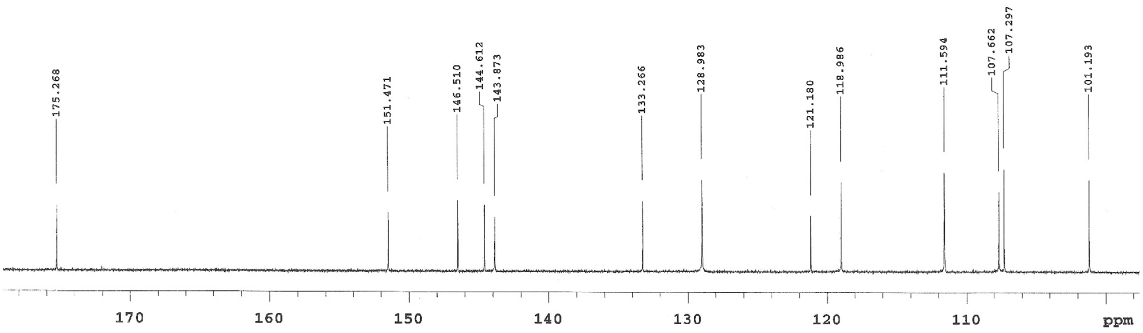

Figure S13. ^{13}C-NMR Spectrum of Compound **4** (101–175 ppm).

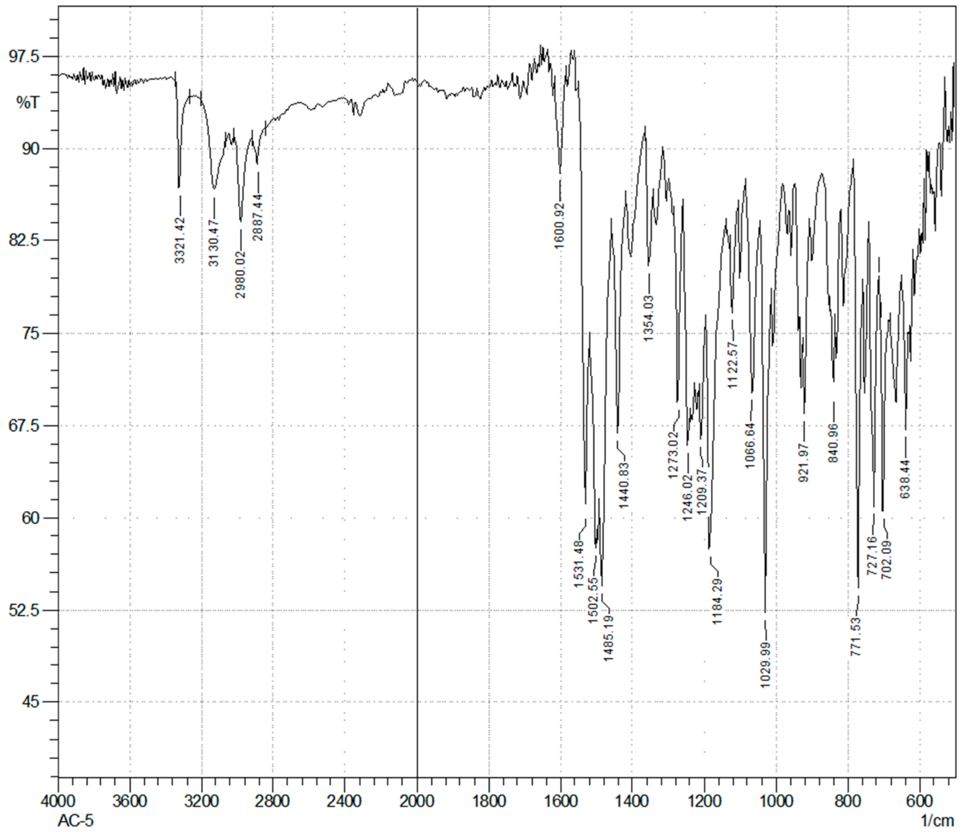

Figure S14. IR Spectrum of Compound **5**.

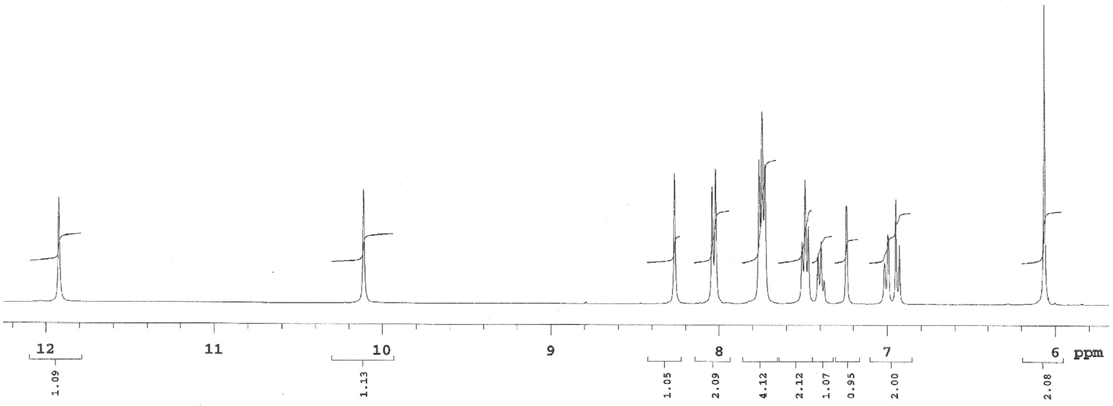

Figure S15. ^{1}H-NMR Spectrum of Compound **5** (6–12 ppm).

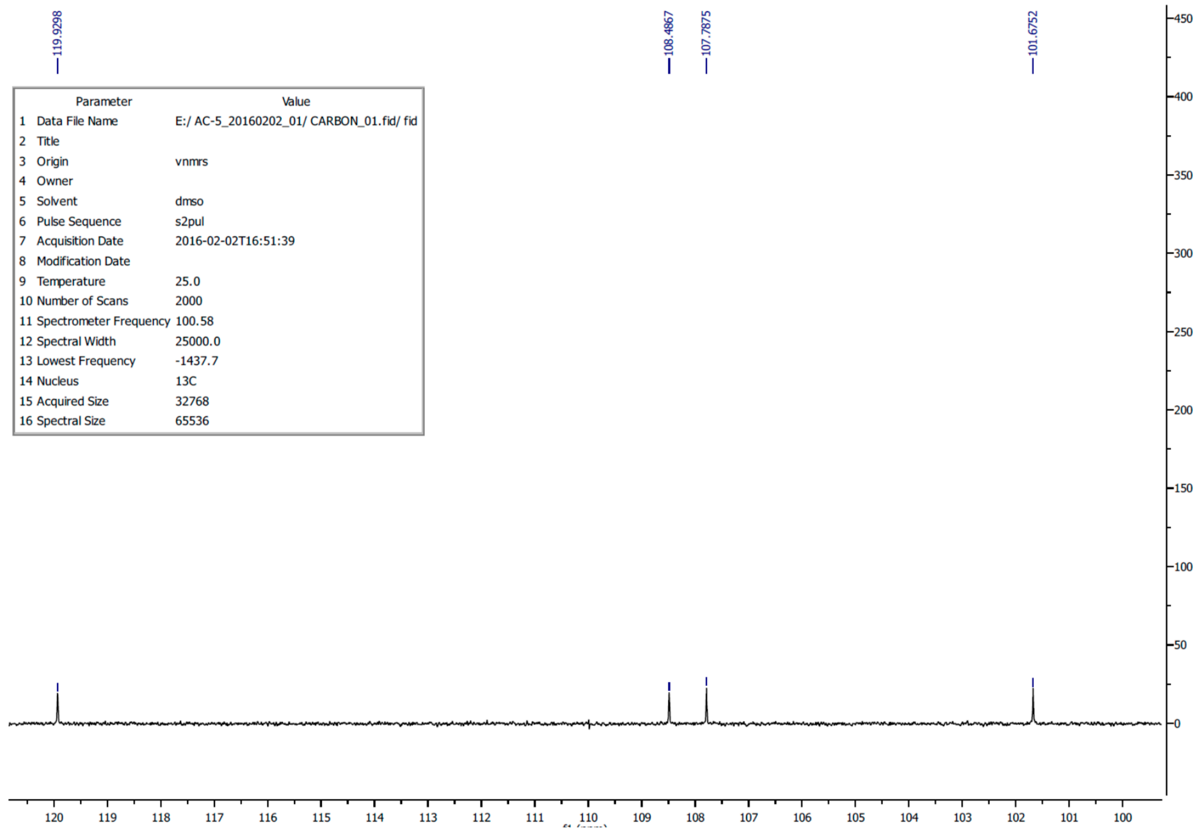

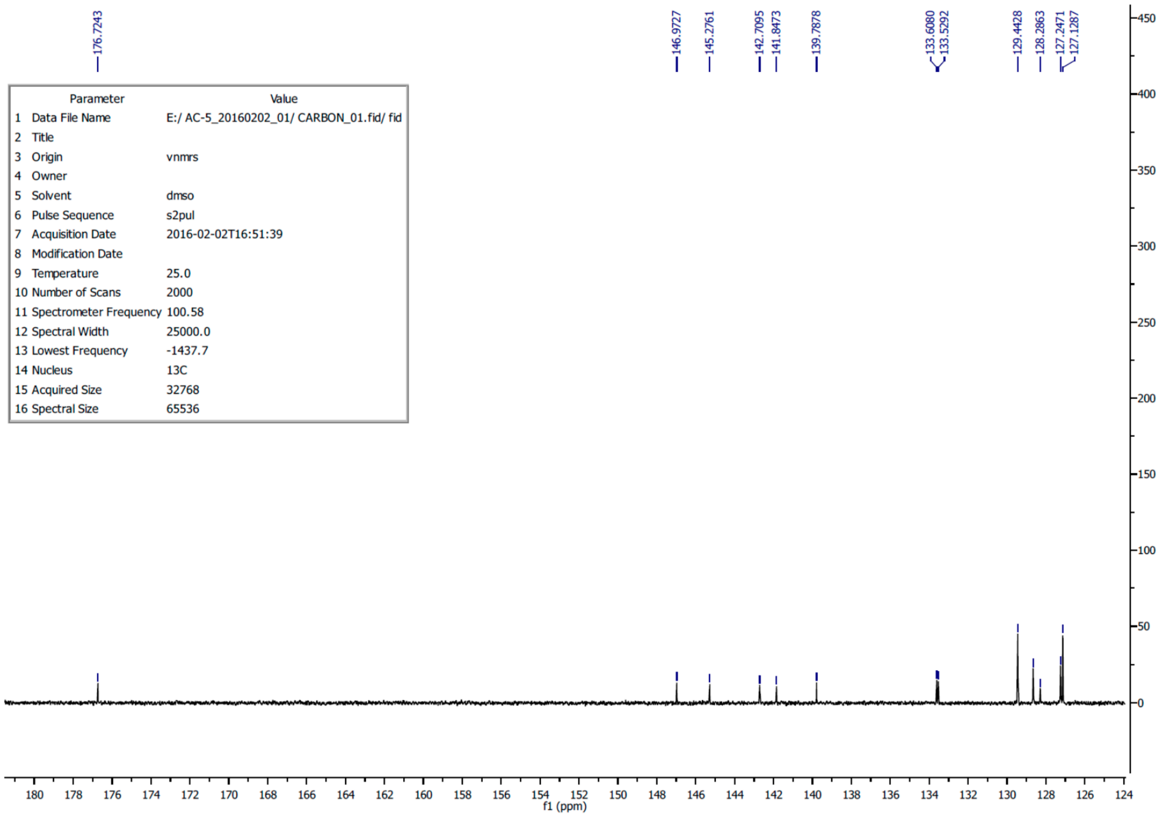

Figure S16. ^{13}C-NMR Spectrum of Compound **5** (101–119, 127–176 ppm).

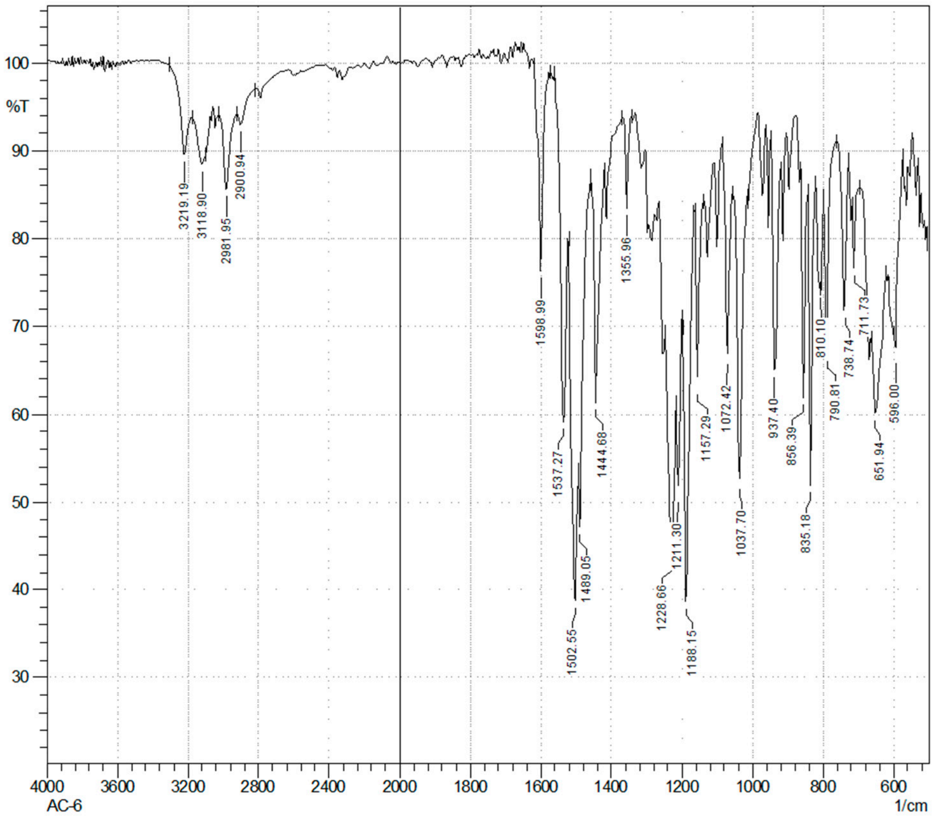

Figure S17. IR Spectrum of Compound **6**.

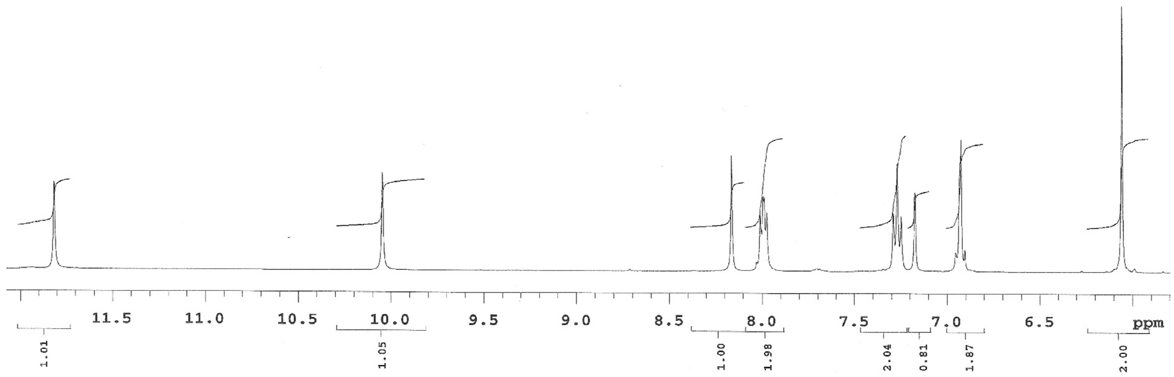

Figure S18. ^{1}H-NMR Spectrum of Compound **6** (6.5–11 ppm).

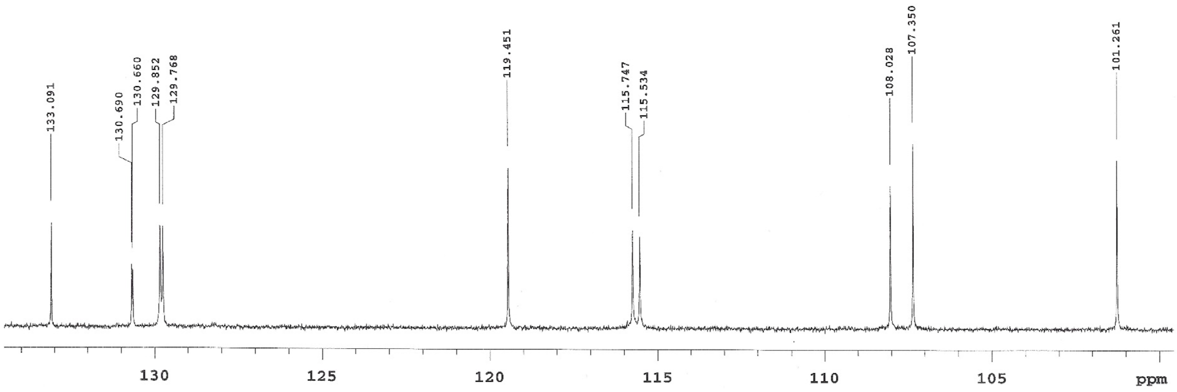

Figure S19. ^{13}C-NMR Spectrum of Compound **6** (101–133 ppm).

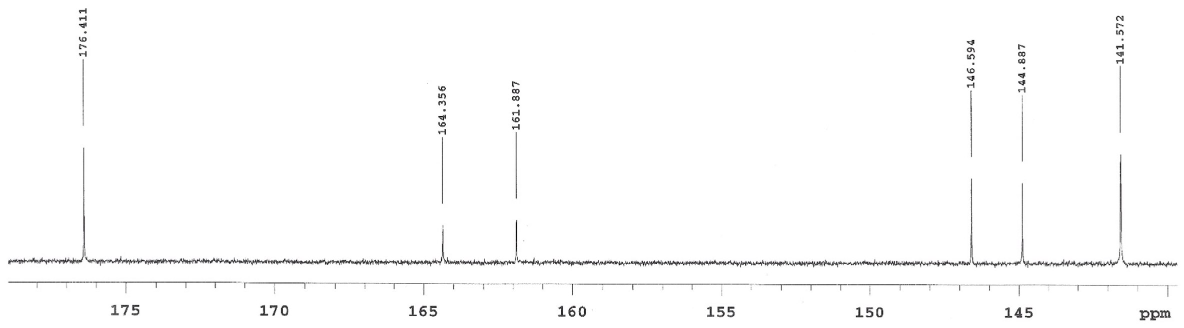

Figure S20. ^{13}C-NMR Spectrum of Compound **6** (141–176 ppm).

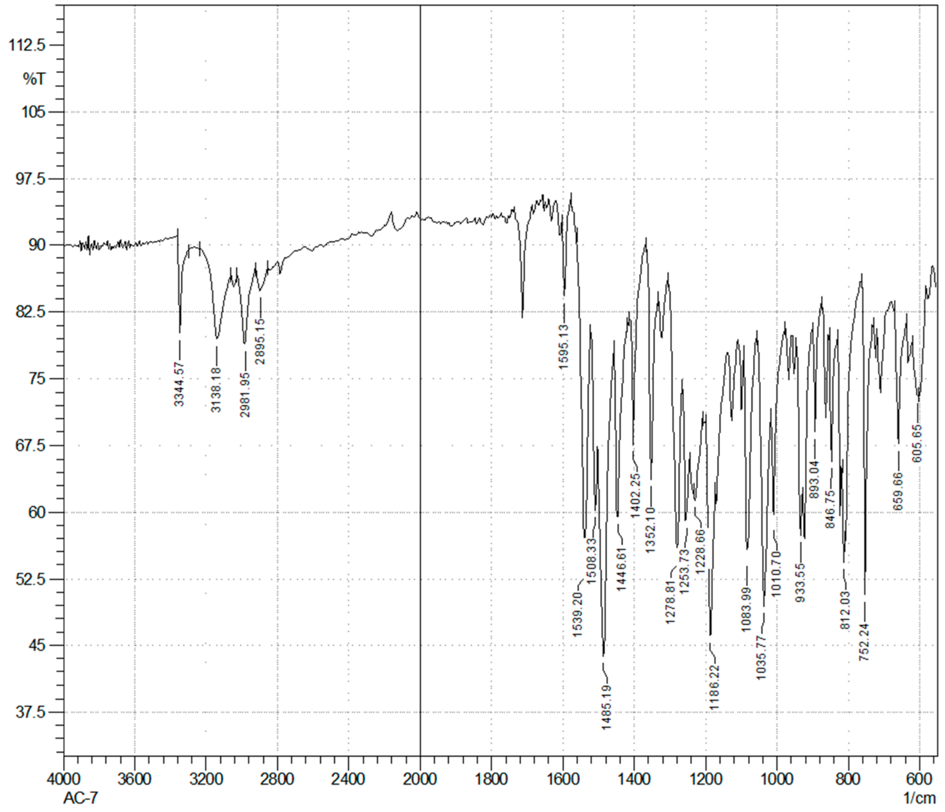

Figure S21. IR Spectrum of Compound **7**.

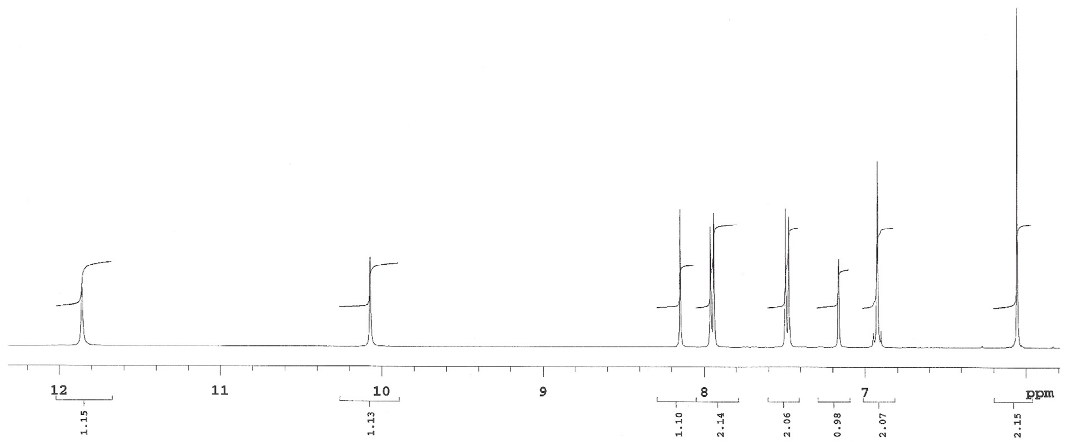

Figure S22. ^{1}H-NMR Spectrum of Compound **7** (7–12 ppm).

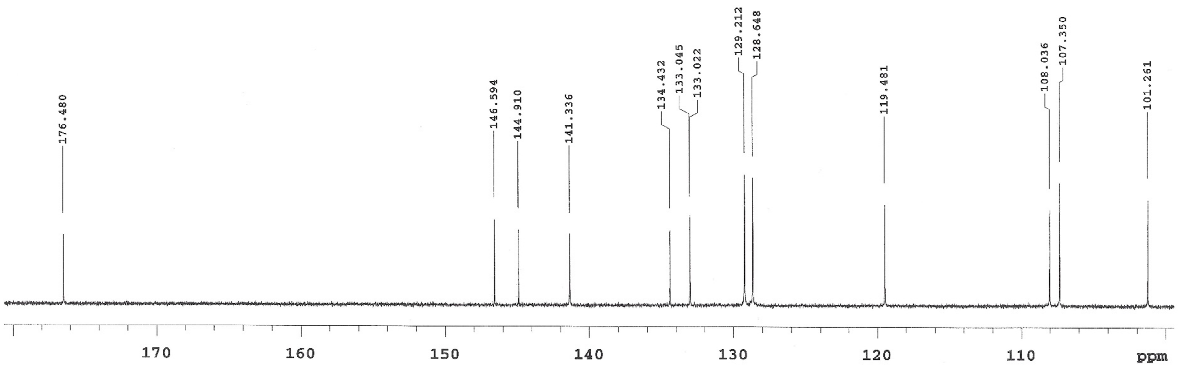

Figure S23. ^{13}C-NMR Spectrum of Compound **7** (101–176 ppm).

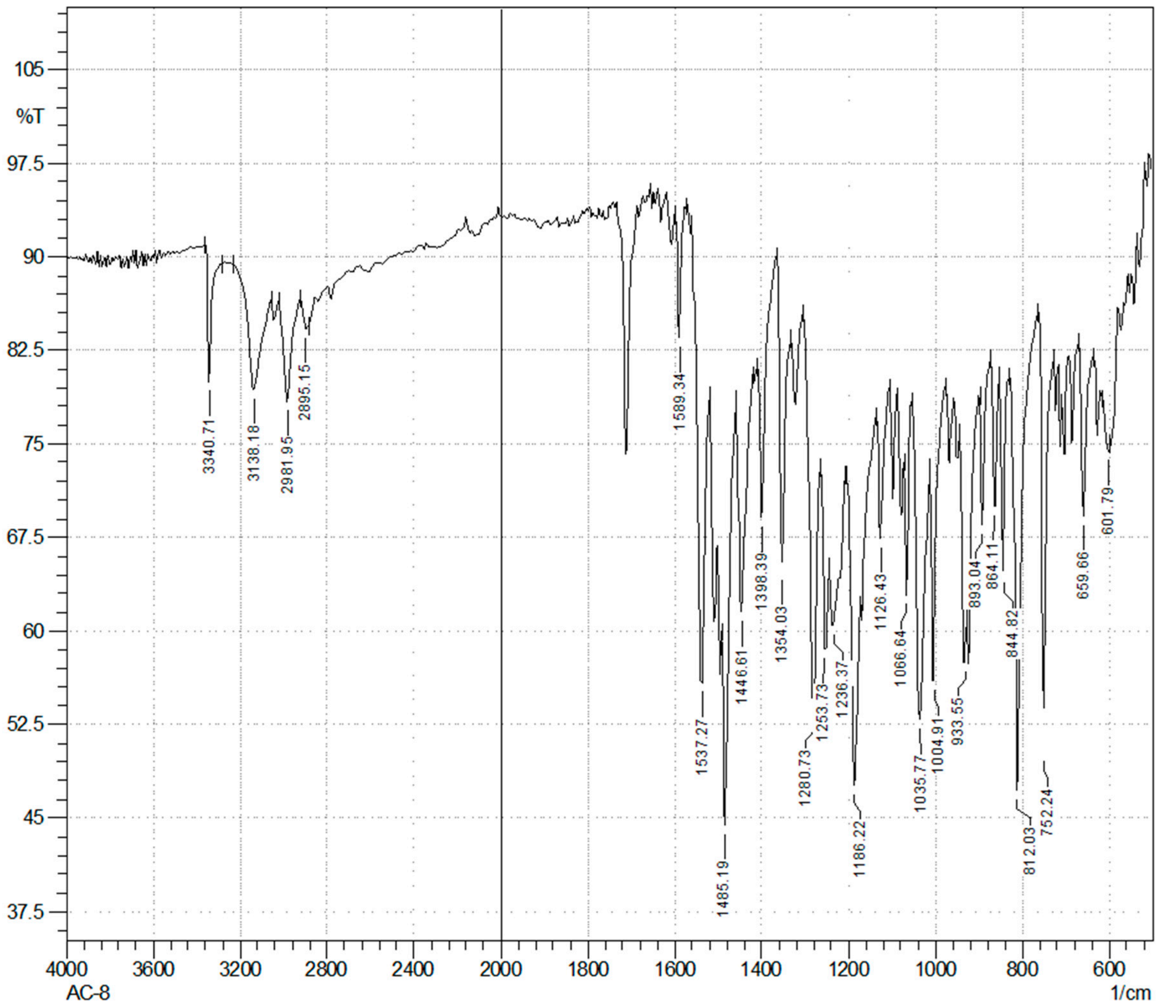

Figure S24. IR Spectrum of Compound **8**.

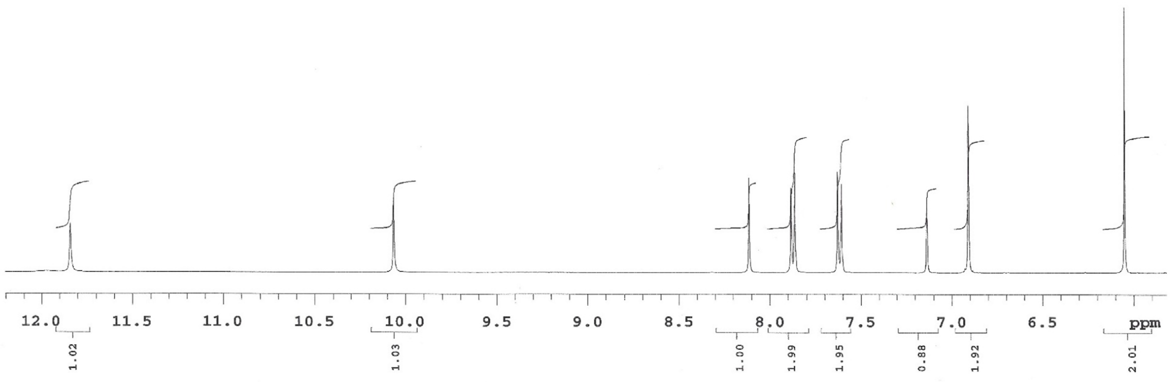

Figure S25. ^{1}H-NMR Spectrum of Compound **8** (6.5–12 ppm).

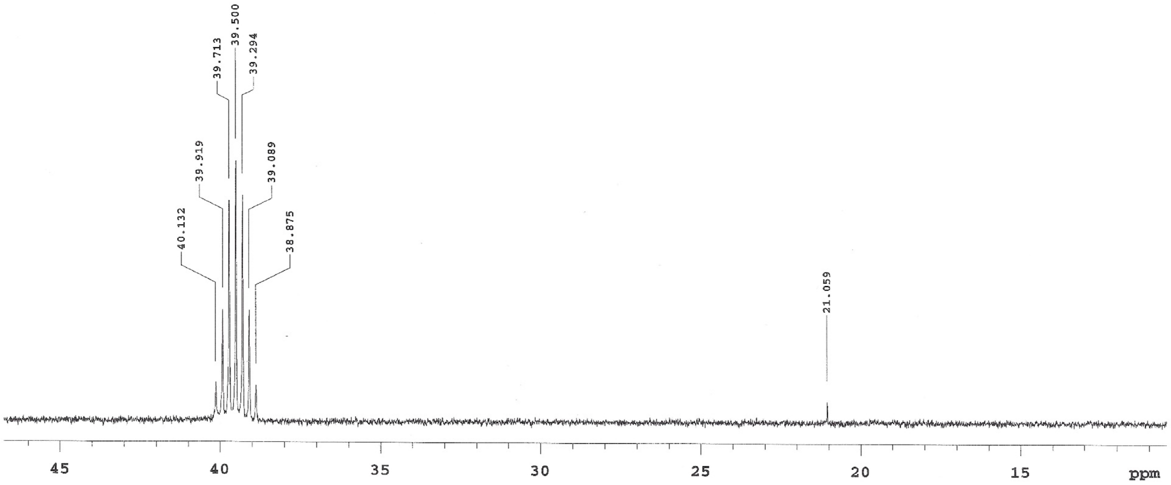

Figure S26. ^{13}C-NMR Spectrum of Compound **8** (20–45 ppm).

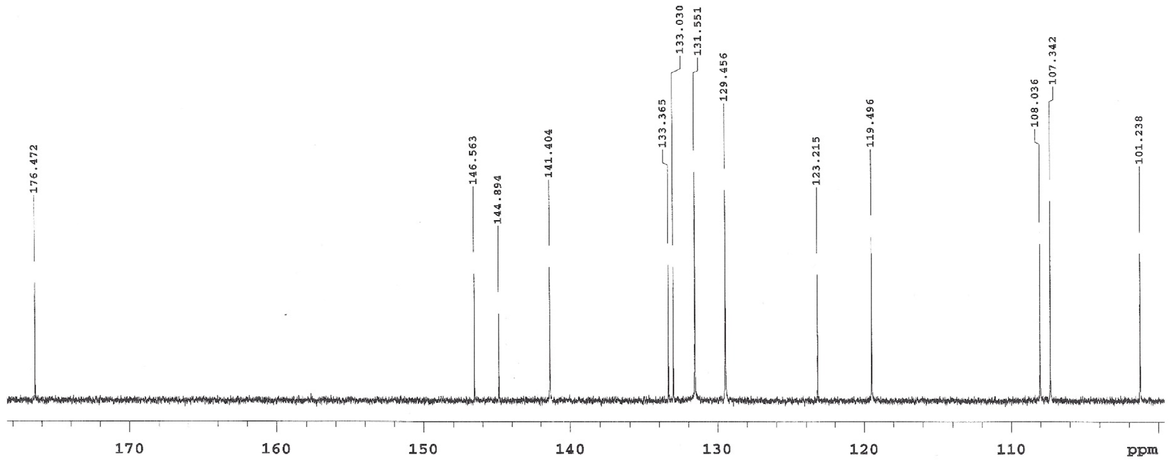

Figure S27. ^{13}C-NMR Spectrum of Compound **8** (101–176 ppm).

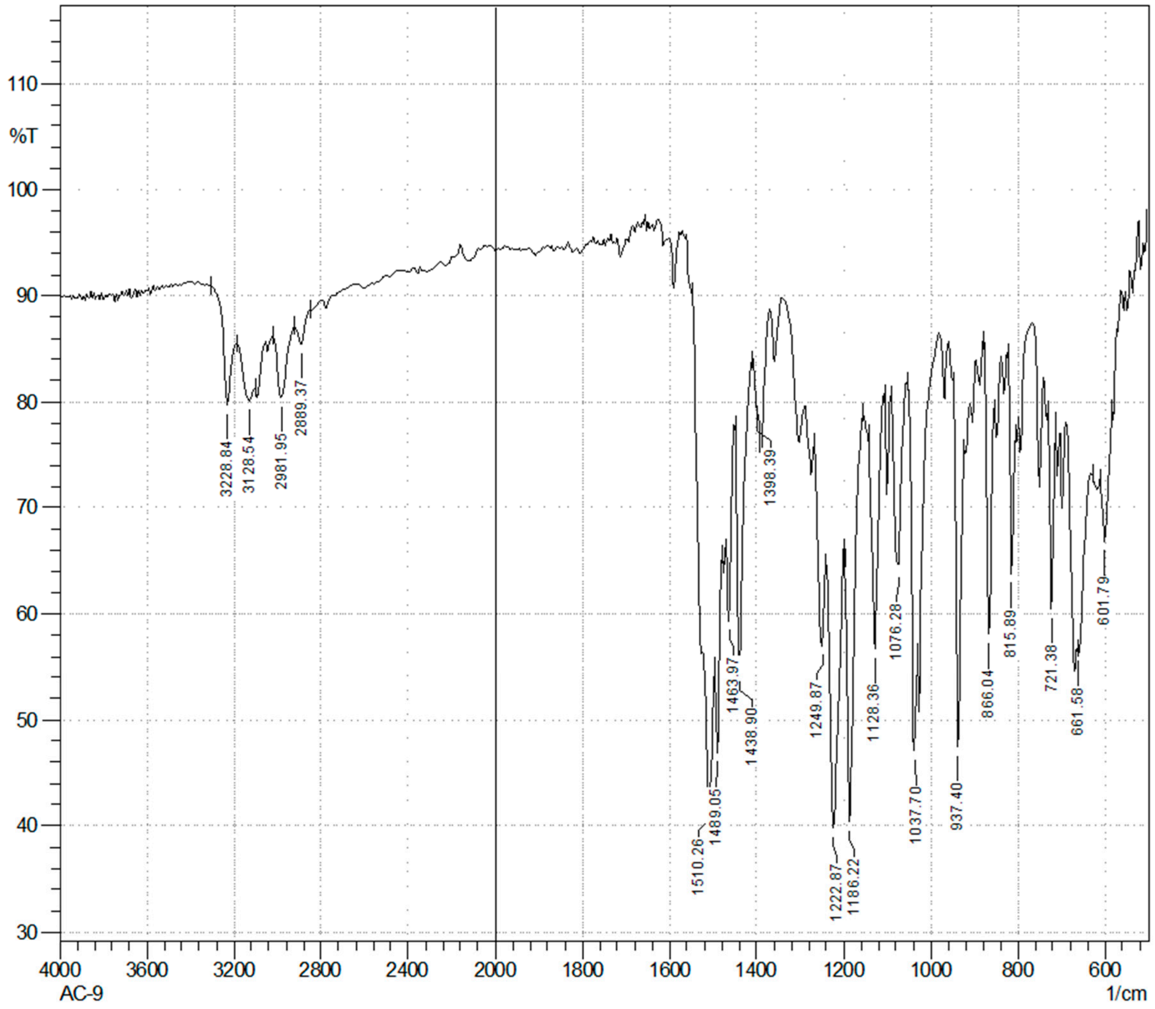

Figure S28. IR Spectrum of Compound **9**.

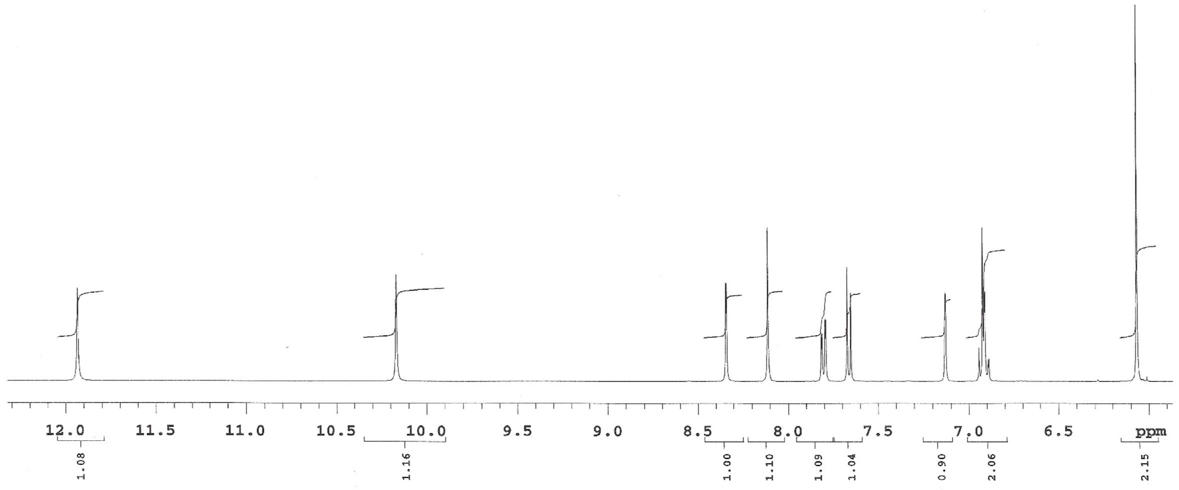

Figure S29. ^{1}H-NMR Spectrum of Compound **9** (6.5–12 ppm).

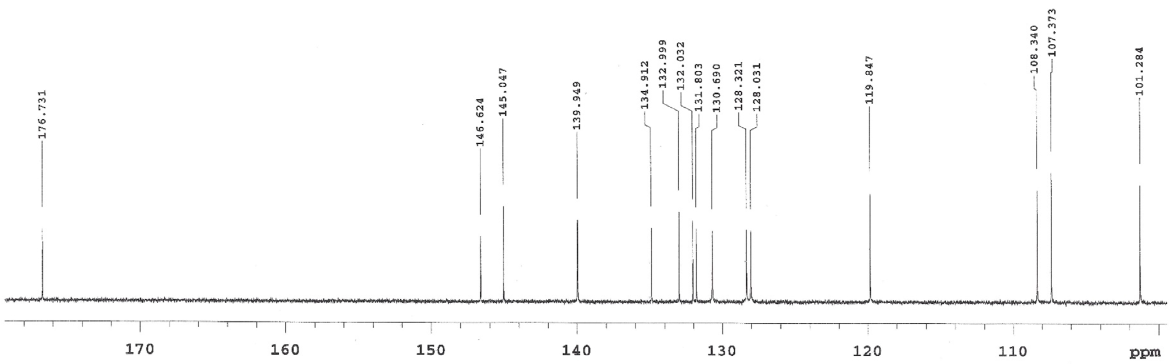

Figure S30. ^{13}C-NMR Spectrum of Compound **9** (101–176 ppm).

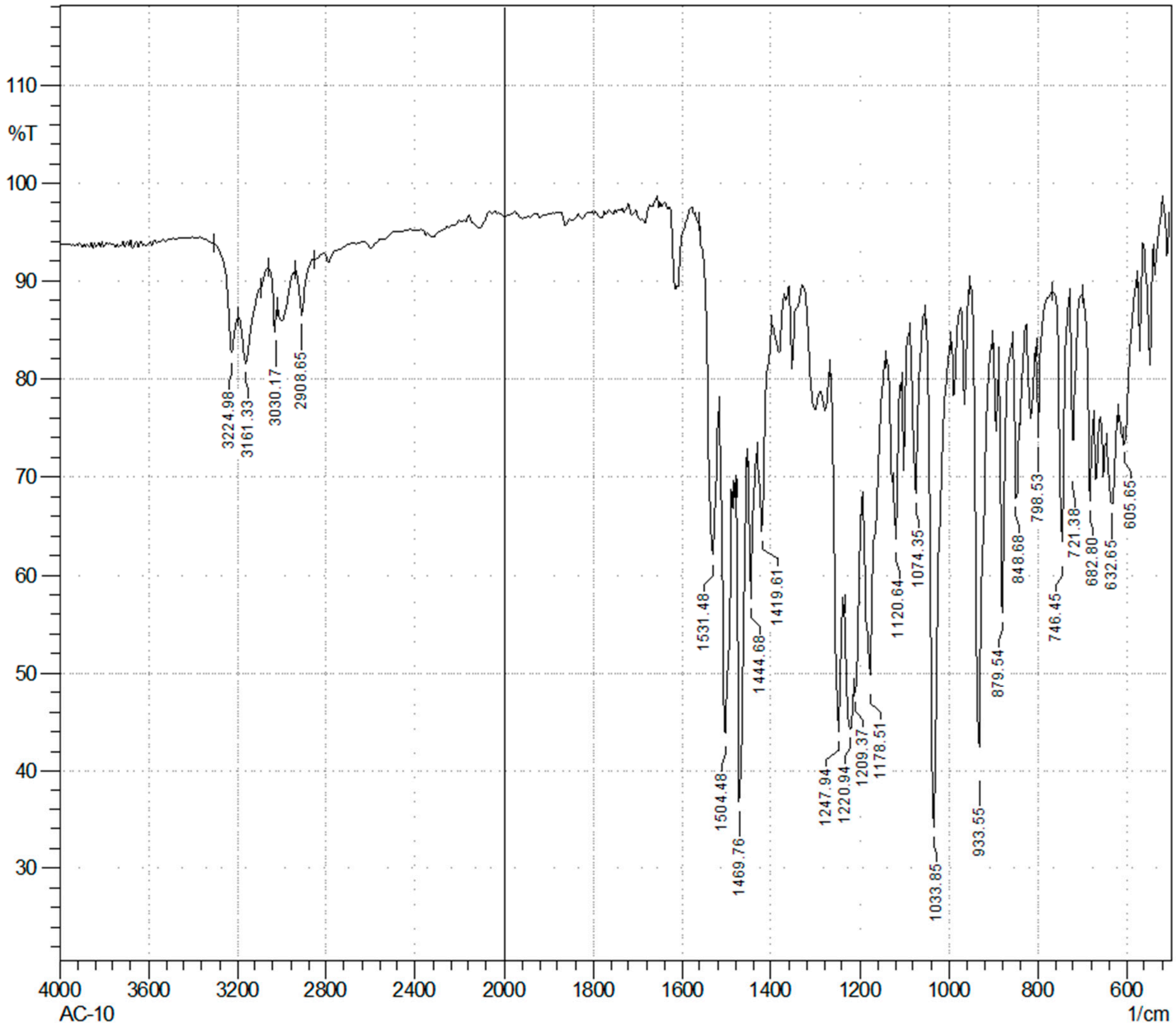

Figure S31. IR Spectrum of Compound **10**.

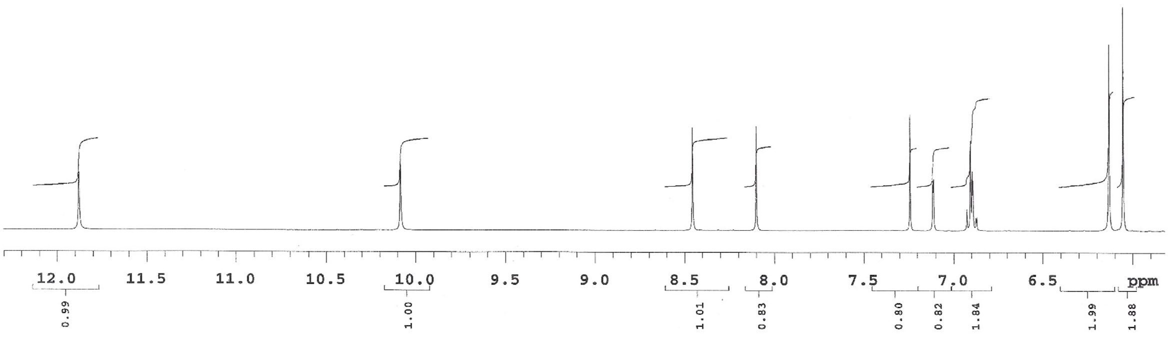

32. ^{1}H-NMR Spectrum of Compound **10** (6.5–12 ppm)

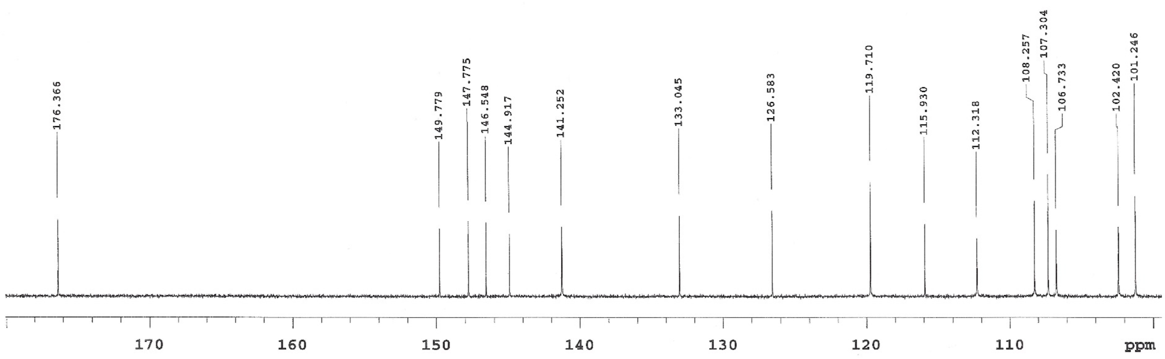

Figure 33. ^{13}C-NMR Spectrum of Compound **10** (101–176 ppm).
